# Preventing BRCA1/ZBRK1 repressor complex binding to the *GOT2* promoter results in accelerated aspartate biosynthesis and promotion of cell proliferation

**DOI:** 10.1002/1878-0261.12466

**Published:** 2019-03-01

**Authors:** Ruoxi Hong, Weimin Zhang, Xi Xia, Kai Zhang, Yan Wang, Mengjiao Wu, Jiawen Fan, Jinting Li, Wen Xia, Fei Xu, Jie Chen, Shusen Wang, Qimin Zhan

**Affiliations:** ^1^ State Key Laboratory of Oncology in South China Collaborative Innovation Center for Cancer Medicine Sun Yat‐sen University Cancer Center Guangzhou China; ^2^ Key Laboratory of Carcinogenesis and Translational Research (Ministry of Education/Beijing) Laboratory of Molecular Oncology Peking University Cancer Hospital & Institute Beijing China; ^3^ Beijing Advanced Innovation Center for Food Nutrition and Human Health College of Veterinary Medicine China Agricultural University Beijing China; ^4^ Beijing Key Laboratory of Detection Technology for Animal‐Derived Food Safety and Beijing Laboratory for Food Quality and Safety China Agricultural University Beijing China; ^5^ State Key Laboratory of Molecular Oncology National Cancer Institute and Cancer Hospital Chinese Academy of Medical Sciences and Peking Union Medical College Beijing China

**Keywords:** aspartate, BRCA1, breast cancer, co‐repressor, GOT2, ZBRK1

## Abstract

Breast cancer susceptibility gene 1 (BRCA1) has been implicated in modulating metabolism via transcriptional regulation. However, direct metabolic targets of BRCA1 and the underlying regulatory mechanisms are still unknown. Here, we identified several metabolic genes, including the gene which encodes glutamate‐oxaloacetate transaminase 2 (GOT2), a key enzyme for aspartate biosynthesis, which are repressed by BRCA1. We report that BRCA1 forms a co‐repressor complex with ZBRK1 that coordinately represses GOT *2* expression via a ZBRK1 recognition element in the promoter of *GOT2*. Impairment of this complex results in upregulation of GOT2, which in turn increases aspartate and alpha ketoglutarate production, leading to rapid cell proliferation of breast cancer cells. Importantly, we found that GOT2 can serve as an independent prognostic factor for overall survival and disease‐free survival of patients with breast cancer, especially triple‐negative breast cancer. Interestingly, we also demonstrated that GOT2 overexpression sensitized breast cancer cells to methotrexate, suggesting a promising precision therapeutic strategy for breast cancer treatment. In summary, our findings reveal that BRCA1 modulates aspartate biosynthesis through transcriptional repression of GOT2, and provides a biological basis for treatment choices in breast cancer.

**Abbreviations**
AJCC: American Joint Committee on Cancer
BC: breast cancer
BRCA1: breast cancer susceptibility gene 1
ChIP: chromatin immunoprecipitation
GOT2: glutamate oxaloacetate transaminase 2
HR: Hazard ratio
IHC: immunohistochemistry
LNM: lymph node metastasis
MTX: methotrexate
TNBC: triple‐negative breast cancer
ZBRK1: zinc finger and BRCA1‐interacting protein with a KRAB domain 1
α‐KG: α‐ketoglutaric acid (2‐oxoglutaric acid)

## 1 Introduction

Metabolic reprogramming is a well‐recognized hallmark of cancer (Hanahan and Weinberg, 2011). To sustain rapid proliferation and survive in a nutrient‐poor environment, tumor cells have evolved a sophisticated adaptive metabolic signaling to generate ATP as energy source, maintain redox balance and commit resources to biosynthesis, including increased glycolysis, glutaminolysis and lipid synthesis (Hirschey *et al*., 2015; Martinez‐Outschoorn *et al*., 2017). Moreover, tumor cells arising in different tissues or even within the same tissue have different metabolic profiles, even when they have identical genetic lesions, suggesting that tumor cells rewire their metabolic pathways to adapt to different tissue environments or tumor microenvironments (TME) (Anastasiou, 2017; Danhier *et al*., 2017; Munoz‐Pinedo *et al*., 2012; Yuneva *et al*., 2012). Although metabolic variability and flexibility support tumor progression, they also render tumor cells more susceptible to perturbations within the metabolic network. However, the dynamic regulatory processes and heterogeneity of tumor cell metabolism are far from being understood. Thus, investigating the metabolic dependency of tumor cells may open a promising new avenue for therapeutic intervention.

Breast cancer susceptibility gene 1 (*BRCA1*) is a major breast cancer suppressor gene, which is most frequently mutated in hereditary breast cancer and is often downregulated in sporadic breast cancer (Miki *et al*., 1994; Yarden and Papa, 2006). The BRCA1 protein is a pluripotent regulator of cellular functions in breast cancer, including DNA double‐strand break repair, cell cycle control, transcriptional regulation, ubiquitination, apoptosis and resistance to anticancer agents (Silver and Livingston, 2012). There is growing evidence linking BRCA1 to metabolic reprogramming. BRCA1 has been shown to interact with many key metabolic regulators, including p53, Myc, Akt (Xiang *et al*., 2008), HIF‐1α (van der Groep *et al*., 2008; Kang *et al*., 2006), Oct1 (Vazquez‐Arreguin *et al*., 2018) and acetyl‐CoA carboxylase (Moreau *et al*., 2006). Maud Privat and colleagues have revealed that BRCA1 inhibited glycolysis while activating TCA cycle and oxidative phosphorylation through global transcriptional and metabolite profiling (Privat *et al*., 2014). Except for these correlations, the casual roles and underlying molecular mechanisms of BRCA1 in metabolic adaptation are still unclear, especially for the transcriptional regulation functions of BRCA1 in metabolism. Because BRCA1 lacks the ability to recognize these regulatory sequences, it must associate with sequence‐specific binding transcription factors to execute its transcriptional function (Yun and Lee, 2003). Zinc finger and BRCA1‐interacting protein with KRAB domain‐1 (ZBRK1, also known as ZNF350), which was first identified in a yeast two‐hybrid screening for proteins associated with BRCA1, is a typical KRAB‐containing zinc finger protein that contains a highly conserved KRAB domain at the NH_2_ terminus, and a CTRD domain for BRCA1 interactions at the COOH terminus (Chen *et al*., 1996). Its zinc finger repeats were suggested to recognize a consensus DNA‐binding element, GGGxxxCAGxxxTTT, or to be involved in protein interactions (Bellefroid *et al*., 1991). BRCA1 transcriptionally repressed the expression of several genes by forming a complex with ZBRK1, which including *GADD45A* (Zheng *et al*., 2000), *HMGA2* (Ahmed *et al*., 2010), *MMP9* (Lin *et al*., 2010) and *ANG1* (Furuta *et al*., 2006). However, whether ZBRK1 plays a direct role in BRCA1‐associated metabolism reprogramming has yet to be elucidated.

In the present study, we sought to determine the metabolic target genes of BRCA1/ZBRK1 transcriptional repressor complex. To this end, by integrating mRNA microarray profiles and bioinformatics analysis, we identified glutamate‐oxaloacetate transaminase 2 (GOT2), a key enzyme for aspartate production, as a direct transcriptionally repressed target of BRCA1/ZBRK1. Our findings revealed that BRCA1/ZBRK1 complex could have a function in aspartate metabolism, which was partially dependent on transcriptional regulation of *GOT2*. Importantly, GOT2 was overexpressed in breast cancer (BC), especially in triple‐negative breast cancer (TNBC), and was significantly correlated with poor survival of patients with these diseases. Our study also demonstrated that GOT2 was crucial for breast cancer progression and could serve as a predictor of methotrexate (MTX) sensitivity.

## 2 Materials and methods

### 2.1 Analyzing the mRNA microarray data and screening consensus ZBRK1 DNA binding element

Previous mRNA microarray profiles of BRCA1‐deleted mouse embryonic fibroblast MEF‐BRCA1^▵/▵^ and the wild‐type counterparts (MEF‐BRCA1^+/+^) were used for analysis, specifically focusing on metabolic enzymes and transporters. The differential expression genes were defined by the relative expression fold change (MEF‐BRCA1^▵/▵^ vs MEF‐BRCA1^+/+^) > 2 or < 0.5.

For consensus ZBRK1 DNA binding element screening, 2000‐bp upstream sequences of the transcriptional start site of each metabolic gene were downloaded from UCSC Genome Browser (https://genome.ucsc.edu/cgi-bin/hgTables). Consensus ZBRK1 DNA binding element GGxxxCAGxxxT was searched for in these sequences by r software.

### 2.2 Cell lines and cell culture

Human breast cancer cell lines MDA‐MB‐231, MCF‐7, CAL‐51, MDA‐MB‐468, human embryonic kidney cell line HEK293T and mouse embryonic fibroblast MEF‐BRCA1^▵/▵^ and the wild‐type counterparts MEF‐BRCA1^+/+^ (kindly provided by Professor Chuxia Deng of National Institute of Diabetes, Digestive, and Kidney Diseases) were maintained in Dulbecco's modified Eagle's medium (DMEM, Thermo Fisher Scientific, New York, NY, USA) supplemented with 10% FBS. For aspartate or alpha ketoglutarate (α‐KG) rescue assay, cells were treated with 10 μm aspartate or 2 mm α‐KG for the indicated times. To inhibit transaminases, cells were treated with 1 mm aminooxyacetic acid hemihydrochloride (AOA) from MedChemExpress (HY‐107994) in complete medium for 48 h. All of these cells were maintained at 37 °C with 5% CO_2_.

### 2.3 RNA isolation and quantitative real‐time PCR

Total RNA of breast cancer cells was extracted with TRIzol reagent (Invitrogen, Carlsbad, CA, USA). The cDNA was synthesized with the PrimeScript RT reagent Kit (Promega, Madison, WI, USA). Real‐time PCR was carried out using an ABI 7500 real‐time PCR system (Applied Biosystems, Foster City, CA, USA). The mRNA expression level of GOT2 and ASS1 were normalized to the endogenous expression of GAPDH. Primers were provided by Invitrogen as described below:

GAPDH forward, 5′‐TCTCTGCTCCTCCTGTTC‐3′,
GAPDH reverse, 5′‐GTTGACTCCGACCTTCAC‐3′;
GOT2 forward, 5′‐GAGCAGGGCATCAATGTCTG‐3′
GOT2 reverse, 5′‐GTTGGAATACAGGGGACGGA‐3′
ASS1 forward, 5′‐TGTGCTTATAACCTGGGATGGG‐3′
ASS1 reverse, 5′‐GACATAGCGTCTGGGATTGGA‐3′

### 2.4 Plasmid construction, transfection and lentivirus infection

Plasmid pcDNA3.1‐BRCA1 and pcDNA3.1‐ZBRK1 (also known as ZNF350) were constructed by inserting the corresponding human cDNA into the *Hind*III/*Xho*I sites of pcDNA3.1 vector. The region −929 to −130 of GOT2 promoter, which containing the consensus ZBRK1 DNA binding element (ZBE) GGGaggCAGaggTTG, was inserted in the *Nhe*I/*Xho*I sites of pGL3‐basic plasmid, hereafter termed GOT2 promoter Wt. The mutant of GOT2 promoter (GOT2 promoter Mut) was constructed by deleting the ZBE of GOT2 promoter Wt. After constructing pLVX‐GOT2 by inserting the corresponding human cDNA into the *Eco*RI/*Bam*HI sites of pLVX‐puro vector, pLVX‐GOT2 was packaged into the lentivirus system with pMD2.G and pSPAX2 vectors using HEK293T cells. Lentiviral shRNA of GOT2 were purchased from GeneChem Co. Ltd. Shanghai, China. The GOT2 overexpression or shRNA lentiviruses were infected into cells using polybrene (8 μg·mL^−1^) to enhance infection efficiency. Puromycin (1 μg·mL^−1^) was added to the cells for 72h to screen for stably infected cells.

### 2.5 Luciferase reporter assay

HEK293T cells cultured in 6‐well plates were transiently cotransfected with different plasmid combinations as below and internal control plasmid, using the Lipofectamine 2000 Reagent according to the manufacturer's protocol (Thermo Fisher Scientific, Carlsbad, CA, USA):

Different amounts of pcDNA3.1‐BRCA1 (0, 2, 4 μg) with 2 μg GOT2 promoter Wt

Different amounts of pcDNA3.1‐ZBRK1 (0, 2, 4 μg) with 2 μg GOT2 promoter Wt

2 μg pcDNA3.1 vector with 2 μg GOT2 promoter Wt or GOT2 promoter Mut

2 μg pcDNA3.1‐BRCA1 with 2 μg GOT2 promoter Wt or GOT2 promoter Mut

2 μg pcDNA3.1‐ZBRK1 with 2 μg GOT2 promoter Wt or GOT2 promoter Mut

Forty‐eight hours post‐transfection, cells were lysed in passive lysis buffer, and luciferase assay was performed using a Dual‐Luciferase Reporter Assay kit (Promega). Renilla luciferase activity was used as control for the transfection. Each transfection was performed in triplicate.

### 2.6 Western blot

Cells were harvested and lysed on ice for 40 min in RIPA buffer (10 mm Tris pH 7.4, 150 mm NaCl, 1% Triton X, 0.1% Na‐deoxycholate, 0.1% SDS and 5 mm EDTA) containing Complete Protease Inhibitor Cocktail (Roche Applied Science, Indianapolis, IN, USA). The concentration of cellular whole protein was quantified by Pierce™ BCA protein assay kit (Thermo Fisher Scientific, New York, NY, USA). A 20‐μg aliquot of whole proteins was separated by 8% or 10% SDS/PAGE gel and then transferred to PVDF membrane. After blocking with 2% BSA, the membrane was incubated with primary antibodies overnight at 4 °C. The antibodies used were antibodies to BRCA1 (Cell Signaling, Boston, MA, USA), ZBRK1 (Abcam, Cambridge, UK), GOT2 (Abcam; Proteintech Group, Wuhan, China), β‐actin (Sigma, St. Louis, MO, USA), ASS1 (Proteintech Group), GPT2 (Proteintech Group), GDH1 (Proteintech Group). Secondary antibodies such as goat anti‐mouse IgG (1 : 2000) and goat anti‐rabbit IgG (1 : 3000) conjugated with horseradish peroxidase (HRP) were used to probe the membrane for 1 h. The membrane was rinsed in 1 × PBS with 0.1% Tween. After incubation with the chemiluminescence substrate, photographs were taken by GE‐ImageQuant‐LAS‐4000 and Amersham Imager 600 (GE Healthcare Life Sciences, Chicago, IL, USA).

### 2.7 Co‐immunoprecipitation

Cells were lysed with buffer (20 mm Tris/HCL, pH 7.6, 100 mm NaCl, 20 mm KCl, 1.5 mm MgCl_2_, 0.5% NP‐40) containing Complete Protease Inhibitor Cocktail (Roche Applied Science). Cell lysate was incubated with protein A/G‐Sepharose beads preloaded with anti‐BRCA1 or anti‐ZBRK1 antibody. Immunoprecipitates were washed by lysis buffer five times each.

### 2.8 ChIP and ChIP/Re‐ChIP assay

ChIP and ChIP/Re‐ChIP were performed in MCF‐7 cells with a Pierce™ Agarose ChIP Kit (Thermo Fisher Scientific, New York, NY, USA) according to the manufacturer's instructions and using the protocol of Zhang *et al*. (2004, 2006, 2007) as reference. ChIP experiments were conducted in MCF‐7 cells with antibodies against BRCA1 or ZBRK1, and antibody against RNA Polymerase II as positive control. ChIP/Re‐ChIP experiments were conducted in MCF‐7 cells with antibody against BRCA1 in the first round ChIP assay; the eluted DNA was then applied to Re‐ChIP assay with antibody against ZBRK1, and antibody against RNA Polymerase II as positive control. The enrichment of the DNA template was analyzed by real time PCR using primers: forward: 5′‐GGAGGCAGAGGTTGTAGT‐3′ and reverse: 5′‐CACGTCGTTCCTGAATGT‐3′ specific for GOT2 gene promoter, and primers: forward: 5′‐CATGGGTGTGAACCATGAGA‐3′ and reverse: 5′‐GTCTTCTGGGTGGCAGTGAT‐3′ specific for GAPDH gene promoter.

### 2.9 Aspartate, α‐KG and mitochondrial NADH/NAD ^+^ redox measurement

Transfected cells (after 48 h) or MEF‐BRCA1^▵/▵^ and MEF‐BRCA1^+/+^ cells were dissociated and counted. The intracellular levels of aspartate and α‐KG were examined by Aspartate Assay Kit (Abcam) and Alpha Ketoglutarate Assay Kit (Abcam). Briefly, 2 × 10^6^ cells were homogenized in PBS and the supernatant collected. The flow‐through containing the metabolites was used for the measurement of aspartate and α‐KG, following the manufacturer's instructions. The abundance of aspartate and α‐KG were normalized to the cell number. For mitochondrial aspartate, α‐KG and NADH/NAD ^+^ redox detection, mitochondria were separated from 5 × 10^6^ transfected cells or MEF‐BRCA1^▵/▵^ and MEF‐BRCA1^+/+^ cells via commercial kits (Beyotime, Shanghai, China), which has been reported to separate mitochondria from cytosol (Wang *et al*., 2016a). The mitochondria were homogenized in PBS and the supernatant was collected. The flow‐through containing the metabolites was used for the measurement of aspartate, α‐KG and NADH/NAD ^+^ redox (Beyotime), following the manufacturer's instructions.

### 2.10 Cell proliferation and colony formation assay

Cell proliferation was measured by MTS assay kit. Briefly, 3000 cells were seeded in each well of a 96‐well plate and at least four duplicate wells were set for each cell line. From five duplicate 96‐well plates, the relative cell viability by MTS assay kit was measured according to the manufacturer's instructions every day. The relative cell viability (*A*_450__nm_) of Day 0 was measured after cell adherence, and the *A*_450__nm_ of subsequent day points was normalized to the Day 0.

For colony formation assay, 1000 cells were seeded onto 6‐well culture plates and incubated for about 10 days. Culture plates were performed in duplicate. After washing with pre‐cooled PBS, wells were fixed with methanol for 5 min and stained with crystal violet for 10 min. Colonies were examined and automatically calculated by g:box (Syngene).

### 2.11 Ethics statement, tissue specimens and clinicopathological characteristics

Tissue microarrays (TMA) of breast cancer (BC) specimens were obtained from Shanghai Outdo Biotech Co., Ltd. (SOBC; Shanghai, China), with the approval of the Institutional Review Board. The clinicopathological characteristics of all specimens are summarized in Table S5. The samples were collected by surgery in 2001–2004 and were followed up until 2013. An additional 48 paraffin‐embedded TNBC tissue samples of surgical biopsies were obtained, and histopathologically and clinically diagnosed at, the Cancer Institute and Hospital, Chinese Academic of Medical Sciences & Peking Union Medical College (Table S6). Written informed consent was obtained from all patients prior to the study. The use of the clinical specimens for research purposes was approved by the Institutional Research Ethics Committee.

### 2.12 Immunohistochemistry

Immunohistochemistry (IHC) analysis was performed and diagnosed by two pathologists blindly on the 130 paraffin‐embedded BC tissue sections (Tissue Microarray, BC TMA cohort), 90 matched adjacent normal tissues (Tissue Microarray) and the 48 TNBC tissue samples. In brief, the sections were deparaffinized with xylene and rehydrated in graded ethanol. Sections were submerged into EDTA antigenic retrieval buffer (pH 8.0) and microwaved for antigenic retrieval. The sections were then treated with 3% hydrogen peroxide in methanol to quench the endogenous peroxidase activity, followed by incubation with 1% goat serum albumin to block nonspecific binding. The tissue sections were incubated with rabbit anti‐BRCA1 (1 : 200; Cell Signaling), ZBRK1 (1 : 200, Abcam) and GOT2 (1 : 200, Abcam) overnight at 4 °C. After washing, the tissue sections were treated with goat anti‐mouse/rit IgG HRP‐polymer (Dako) for 60 min. 3,3′‐Diaminobenzidine was used as the chromogen.

The scores were determined by combining the intensity of staining and the proportion of positively stained tumor cells as described in our previous paper (1). First, the intensity was graded as follows: 0, negative; 1, weak; 2, moderate; 3, strong. Secondly, the proportion of positive tumor cells was graded: 0 (< 5%), 1 (5–25%), 2 (26–50%), 3 (51–75%) and 4 (> 75%). A final score was derived by multiplication of these two primary scores. Final scores of 0–4 were defined as ‘low expression’ (−) and scores of 6–12 as ‘high expression’ (+). For statistical analysis, 27 additional TNBC clinical data were extracted from the BC TMA cohort and combined with the TNBC cohort for further analysis, termed TNBC cohort hereafter.

### 2.13 Database clinical data analysis

The association of GOT2 mRNA level and BC patient survival was analyzed using the BreastMark database (http://glados.ucd.ie/BreastMark/mRNA_custom.html), KMplot database (http://kmplot.com/analysis/index.php?p=service&cancer=breast) and the Human Protein Atlas database (https://www.proteinatlas.org/).

### 2.14 MTX treatment assay

GOT2 stable knockdown cell line CAL‐51 shGOT2‐1/CAL‐51 shGOT2‐2 and their counterparts, GOT2 stable overexpression cell line MDA‐MB‐468 GOT2 and its counterpart, were used for MTX treatment assay. Briefly, 3000 cells were seeded in each well of a 96‐well plate and at least three duplicate wells were set for each cell line. After 12 h at 37 °C with 5% CO_2_, cells were treated with different concentrations (10, 5, 1, 0.1, 0.01 and 0 μm) of MTX and returned for further incubation for 72 h. MTS assay was used to measure the relative cell viability.

### 2.15 Statistical analysis

Statistical analysis was carried out using ibm spss Statistics 19 (New York, NY, USA) or graphpad prism 5 (San Diego, CA, USA) for Windows. Two‐tailed Student's *t*‐test was used to analyze those results, expressed as mean ± SEM. The two‐tailed Pearson chi‐square test was used to analyze the association of different molecular expression and clinicopathological parameters. Correlation was determined by Pearson's correlation coefficient test or Spearman's rank correlation coefficient analysis. The survival curves were plotted using Kaplan–Meier analysis and compared by log‐rank test. Survival data were also evaluated by multivariate Cox regression analysis. compusyn software was used to calculate the IC_50_. Differences were considered significant when the *P*‐value was less than 0.05.

## 3 Results

### 3.1 BRCA1/ZBRK1 potentially regulates cell metabolism

**Figure 1:**
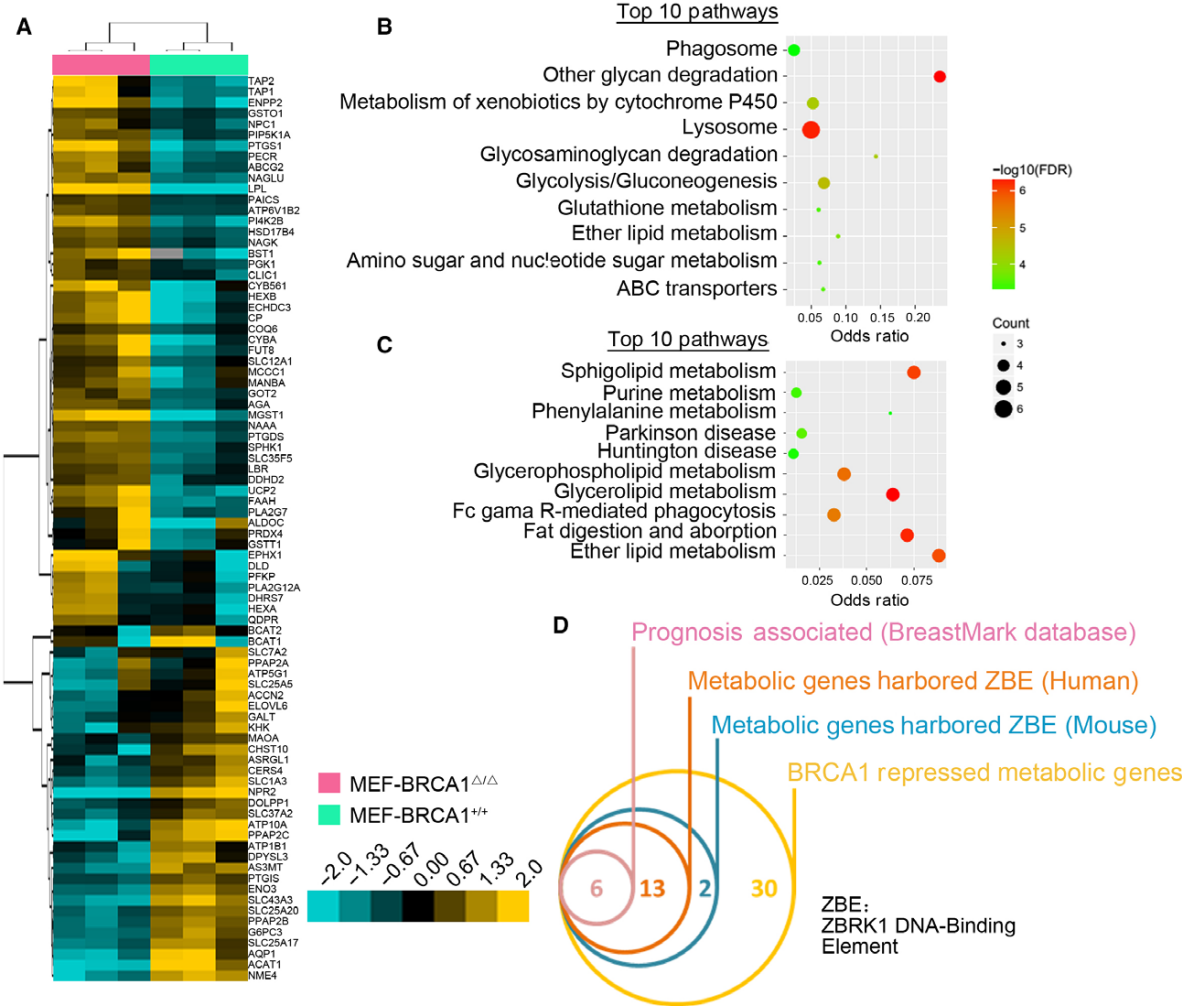
Transcriptional target candidates of BRCA1 and ZBRK1. (A) Heatmap of differentially expressed genes in BRCA1‐deleted mouse embryonic fibroblast MEF‐BRCA1^▵/▵^ and the wild‐type counterparts (MEF‐BRCA1^+/+^). (B) Top 10 metabolism pathways potentially repressed by BRCA1. (C) Top 10 metabolism pathways potentially upregulated by BRCA1. (D) Screening the transcriptional repressed targets of BRCA1/ZBRK1. Promoters of metabolic genes upregulated in MEF‐BRCA1^▵/▵^ cells were used to identify consensus ZBRK1 DNA binding element (ZBE). The genes that harbored ZBE motif both in mouse and human were then evaluated for their correlations with survival outcome via the BreastMark database.

To determine the metabolic pathways regulated by BRCA1, we reanalyzed our previous mRNA microarray profiles of BRCA1‐deleted mouse embryonic fibroblast MEF‐BRCA1^▵/▵^ and the wild‐type counterparts (MEF‐BRCA1^+/+^), with a specific focus on metabolic enzymes and transporters. As expected, a subset of metabolic genes was perturbed by BRCA1 deletion (Fig. 1A, Table S1). Pathway enrichment analysis by genecodis3 (Tabas‐Madrid *et al*., 2012) according to the differential expressed genes revealed that 51 pathways were significantly affected by BRCA1 deficiency (Tables S2 and S3). Thirty‐two pathways were upregulated in MEF‐BRCA1^▵/▵^ cells: e.g. other glycan degradation (*AGA, HEXA, HEXB, MANBA*), glycolysis/gluconeogenesis (*PFKP, DLD, PGK1, ALDOC*), ether lipid metabolism (*PLA2G7, PLA2G12A, ENPP2*) and glutathione metabolism (*MGST1, GSTO1, GSTT1*) (Fig. 1B, Table S2), whereas glycerolipid metabolism (*PPAP2C, PPAP2A, PPAP2B*) and valine, leucine and isoleucine degradation (*ACAT1, BCAT2, BCAT1*) pathways were significantly downregulated in MEF‐BRCA1^▵/▵^ cells (Fig. 1C, Table S3). These findings further confirmed that BRCA1 deficiency could induce metabolism reprogramming.

Given that BRCA1 often serves as a transcriptional co‐repressor through physical interaction with ZBRK1, we sought to determine whether those genes upregulated in MEF‐BRCA1^▵/▵^ cells harbored consensus ZBRK1 DNA‐binding element. Because a previous study has verified that the strict consistency of ZBRK1 DNA‐binding element was not required (Zheng *et al*., 2000), we searched the promoter sequences of BRCA1‐repressed genes with a comparatively strict ZBRK1 DNA‐binding element (GGxxxCAGxxxT); 21 of 51 promoters contained this element (Fig. 1D, Tables S1 and S4). These data indicated that ZBRK1 may be a major mediator in BRCA1‐induced metabolic rewiring. Since the genome is different between human and mouse, we next attempted to determine whether ZBRK1 was also harbored in human genes. Among these candidate BRCA1/ZBRK1‐regulated genes, 19 promoters of human genes contained the GGxxxCAGxxxT elements (Fig. 1D, Table S4), suggesting that these metabolic genes were also regulated by BRCA1/ZBRK1 in human cells.

As BRCA1 and ZBRK1 deficiency contributed to the progression of breast cancer, we supposed that this effect might be partially dependent on its functions in metabolism regulations. Thus, we evaluated the prognostic association of these 19 BRCA1/ZBRK1 candidate repressed genes in breast cancer through the BreastMark database (http://glados.ucd.ie/BreastMark/mRNA_custom.html) (Madden *et al*., 2013). Interestingly, the mRNA levels of six genes (*PGK1, PAICS, GOT2, MGST1, ALDOC* and *PECR*) were significant positively associated with both poor overall survival (OS) and disease‐free survival (DFS) (Table S4). These genes were involved in glycolysis (*PGK1* and *ALDOC*), glutathione metabolism (*MGST1*), aspartate metabolism (*GOT2*), purine metabolism (*PAICS*) and peroxisomal lipid metabolism (*PECR*), indicating that BRCA1/ZBRK1 might play a role in remodeling these metabolic processes through transcriptional regulation of the expression of these genes. We were particularly intrigued by *GOT2*, a key enzyme of malate‐aspartate shuttle, which was critical for cell redox balance and aspartate production. Aspartate is required for rapid cell growth, which is the most important intermediate for protein, purine nucleotide and pyrimidine nucleotide synthesis (Chen *et al*., 1996; Sullivan *et al*., 2015). Dysfunction of BRCA1 is correlated with accelerated growth and progression of breast tumors (Stoppa‐Lyonnet *et al*., 2000; Xu *et al*., 1999). Maintaining redox balance and committing resources to biosynthesis should be essential for the proliferation of BRCA1‐deficient breast cancer cells. Therefore, we focused our interest on BRCA1/ZBRK1‐mediated aspartate metabolism reprogramming through *GOT2* in the present study. The other metabolic pathways are currently being studied by our group.

### 3.2 BRCA1/ZBRK1 suppresses *GOT2* expression

**Figure 2:**
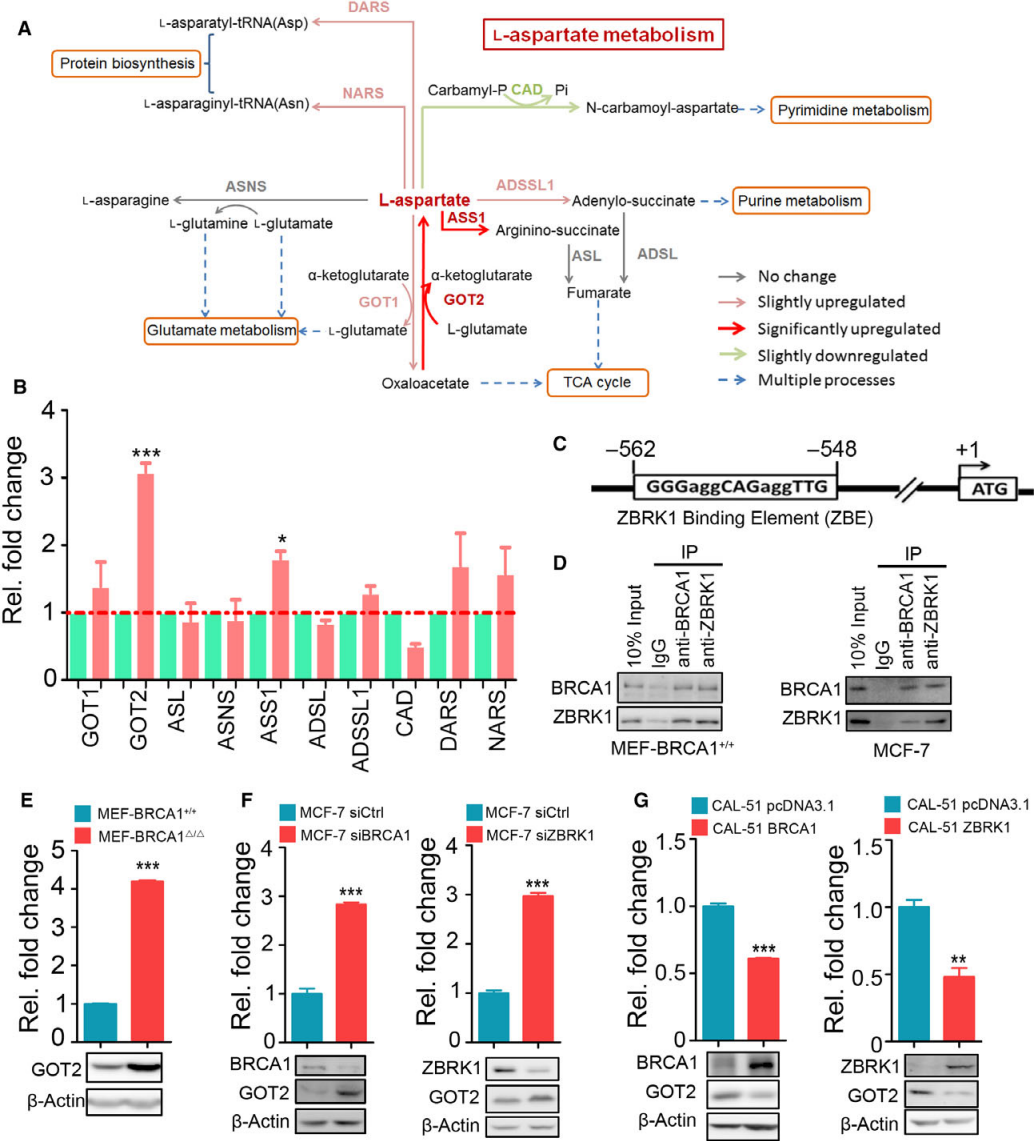
*GOT2* was transcriptionally repressed by BRCA1 and ZBRK1. (A) Schematic representation of aspartate metabolism and its regulation by BRCA1. Genes in red, upregulation; grey, no change; green, downregulation. (B) Relative fold change of key aspartate metabolism genes in MEF‐BRCA1^▵/▵^ cells compared with the wild‐type counterparts MEF‐BRCA1^+/+^ cells. (C) Schematic representation of the promoter of *GOT2*. The region −548 to −562 was the consensus ZBRK1 DNA binding element (ZBE). (D) Co‐IP of BRCA1 and ZBRK1 in MEF‐BRCA1^+/+^ cells (left) and MCF‐7 cells (right). (E) The mRNA (top) and protein level (bottom) of GOT2 in MEF‐BRCA1^▵/▵^ cells and MEF‐BRCA1^+/+^ cells. (F,G) The mRNA (top) and protein level (bottom) of GOT2 in MCF‐7 cells (F) and CAL‐51 cells (G) after knockdown of BRCA1 or ZBRK1 for 48 h. Two‐tailed Student's *t*‐test was used to evaluated the difference; error bars represent the mean ± SEM from three independent experiments. ***P < 0.001; **P < 0.01; *P < 0.05.

Aspartate is a critical metabolic intermediate for protein, purine nucleotide and pyrimidine nucleotide synthesis and is required for cell proliferation (Sullivan *et al*., 2015). We queried whether BRCA1 deficiency could perturb aspartate metabolism. Indeed, the key aspartate biosynthesis enzyme GOT2 and the argininosuccinate synthase 1 (ASS1) were significantly upregulated in BRCA1‐deficient MEF‐BRCA1^▵/▵^ cells, indicating that the aspartate metabolism was tightly regulated by BRCA1 (Fig. 2A,B). Interestingly, the promoter of GOT2, the key enzyme in this pathway, harbored a consensus ZBRK1‐DNA binding element (Fig. 2C), whereas there was no consensus ZBRK1‐DNA binding element found in the promoter of ASS1, suggesting *GOT2* might be coordinately regulated by BRCA1/ZBRK1 repressor complex. To verify this, we first confirmed that BRCA1 indeed physically interacted with ZBRK1 in mouse MEF‐BRCA1^+/+^ cells and human breast cancer cell lines MCF‐7 (Fig. 2D). GOT2 and ASS1 were shown to be significantly upregulated in MEF‐BRCA1^▵/▵^ cells compared with their counterparts in mRNA microarray assay, and this observation was also confirmed by real‐time PCR (Figs 2B,E and S1A). Knockdown of BRCA1 increased, whereas overexpression of BRCA1 decreased the expression of GOT2 and ASS1 at the mRNA and protein level (Figs 2F and S1B). Concordantly, ZBRK1 exerted a similar effect on GOT2; however, knockdown of ZBRK1 did not affect the mRNA or protein levels of ASS1 (Fig. S1C), suggesting that BRCA1 might transcriptionally regulate the expression of ASS1 via an indirect mechanism or other transcription factor other than ZBRK1. Collectively, these findings suggest that BRCA1/ZBRK1 plays a role in repressing *GOT2* expression.

### 3.3 BRCA1 and ZBRK1 form a repressor complex occupied on *GOT2* promoter

**Figure 3:**
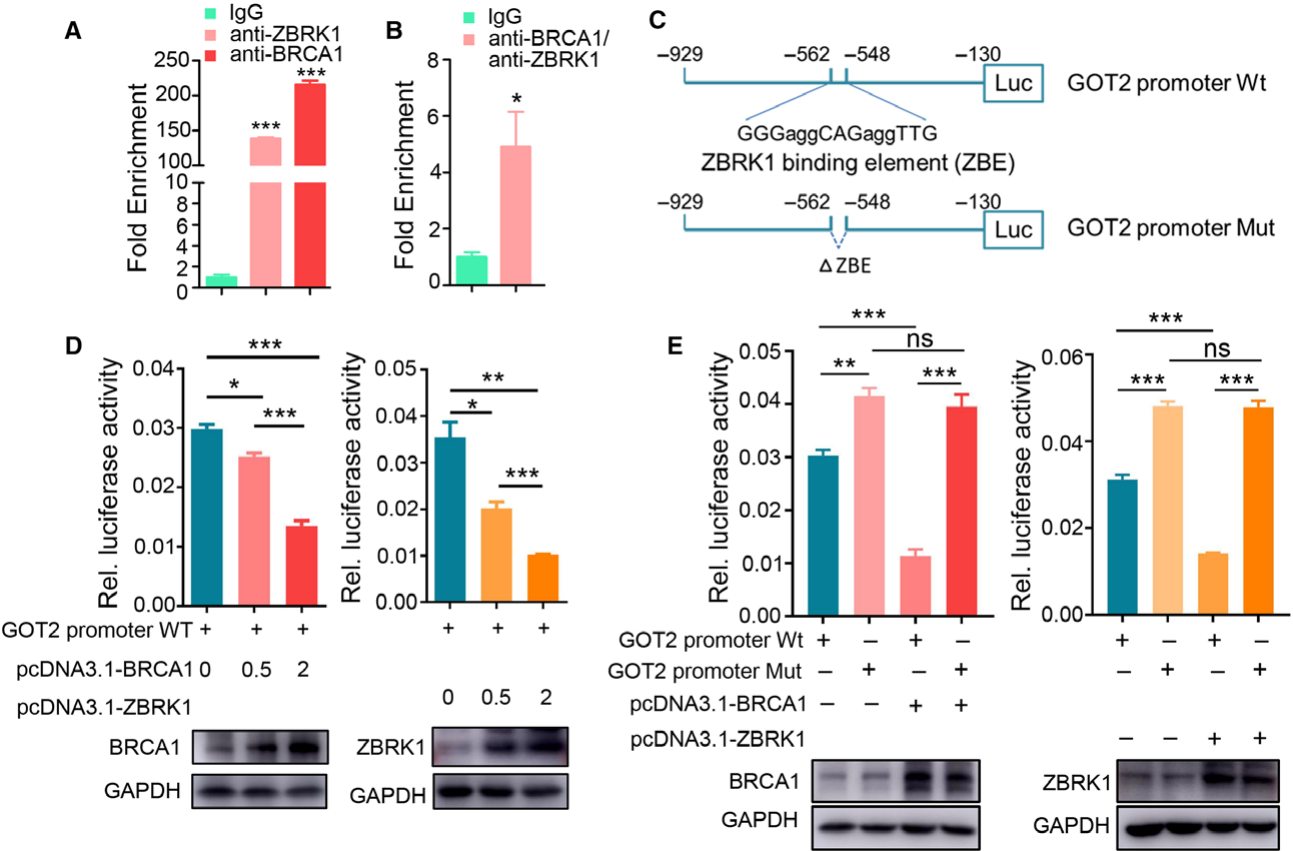
BRCA1 and ZBRK1 co‐repress *GOT2* expression via a single ZBRK1 recognition element in the promoter. (A) Chromatin immunoprecipitation (ChIP) of BRCA1 and ZBRK2 in MCF‐7 cells. Average qPCR results and technical errors (SEM) of the *GOT2* promoter are shown. Fold enrichment was calculated relative to IgG. (B) BRCA1 and ZBRK1 exist in the same protein complex on the *GOT2* promoter. ChIP/Re‐ChIP experiments were performed in MCF‐7 cells with the indicated antibodies. (C) Schematics of *GOT2* reporter constructs in the promoter region. Top, *GOT2* promoter Wt, −929 to −130 region with wild‐type consensus ZBRK1 DNA binding element (ZBE). Bottom, *GOT2* promoter Mut, −929 to −130 region with deletion of ZBE. (D) Relative luciferase activity of reporter constructs GOT2 promoter Wt in HEK293T cells transfected with indicated concentrations of BRCA1 or ZBRK1 plasmids for 48 h. Top, Quantification of relative luciferase activity. Bottom, western blot analysis of ectopic expression of BRCA1 and ZBRK1. (E) Relative luciferase activity of reporter constructs GOT2 promoter Wt and Mut in HEK293T cells transfected with BRCA1 or ZBRK1 plasmids for 48 h. Top, Quantitative of the relative luciferase activity. Bottom, western blot analysis of ectopic expression of BRCA1 and ZBRK1. Two‐tailed Student's *t*‐test was used to evaluated the difference; error bars represent the mean ± SEM from three independent experiments. ***P < 0.001; **P < 0.01; *P < 0.05; ns, not significant.

BRCA1 and ZBRK1 have been shown to form a repressor complex to suppress the expression of target genes. To determine a potential transcriptional regulation of *GOT2* by BRCA1/ZBRK1, we first performed chromatin immunoprecipitation (ChIP) to estimate the physical occupancy of BRCA1 and ZBRK1 within the promoter region of *GOT2*. ChIP experiments were conducted in MCF‐7 cells with antibodies against BRCA1 or ZBRK1, and antibody against RNA polymerase II as positive control (Fig. S2). As expected, BRCA1 and ZBRK1 were both co‐precipitated with the promoter of *GOT2* (Fig. 3A). In addition, ChIP/Re‐ChIP assay (Zhang *et al*., 2004) confirmed that BRCA1 and ZBRK1 exist in the same protein complex on the *GOT2* promoter (Fig. 3B). These experiments not only support the idea that *GOT2* is targeted by the BRCA1/ZBRK1 complex but also confirm that BRCA1 is physically associated with and is an integral component of ZBRK1.

To examine further the transcriptional activity of BRCA1/ZBRK1 on *GOT2*, we measured the luciferase reporter activity of *GOT2* promoter constructs with wild‐type (contained the consensus ZBRK1‐DNA binding element) or mutant (with deletion of the consensus ZBRK1‐DNA binding element, Fig. 3C) after ectopic overexpression of BRCA1 or ZBRK1 (Fig. 3D,E) in HEK293T cells. Only the wild‐type luciferase reporter construct showed a significant decrease in the activity as the expression of BRCA1 or ZBRK1 gradually increased (Fig. 3D); the mutant construct did not show any significant changes (Fig. 3E). These results imply that this region (−548 to −562) is essential for transcriptional repression by BRCA1/ZBRK1.

### 3.4 BRCA1/ZBRK1 modulates aspartate metabolism through repression of *GOT2* expression

**Figure 4:**
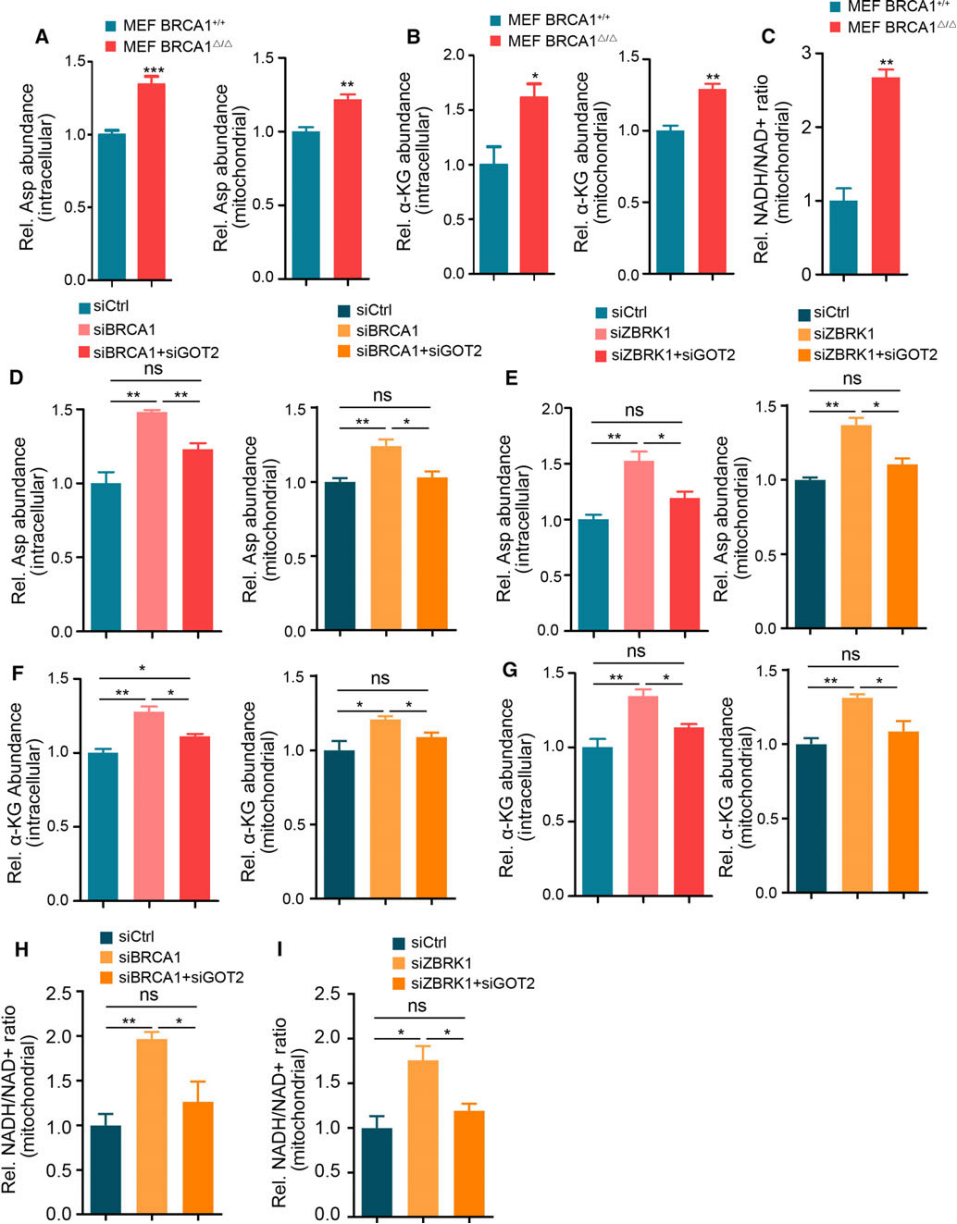
BRCA1/ZBRK1 modulated aspartate and α‐KG production. (A,B) The relative abundance of intracellular (right) and mitochondrial aspartate (A) and α‐KG (B) in MEF‐BRCA1^▵/▵^ cells and MEF‐BRCA1^+/+^ cells. (C) The relative mitochondrial NADH/NAD ^+^ ratio in MEF‐BRCA1^▵/▵^ cells and MEF‐BRCA1^+/+^ cells. (D,E) The relative abundance of intracellular and mitochondrial aspartate in MCF‐7 cells after transfection with *BRCA1* (D) or *ZBRK1* (E) RNAi with or without *GOT* *2* RNAi simultaneously for 48 h. (F,G) The relative abundance of intracellular and mitochondrial α‐KG in MCF‐7 cells after transfection with *BRCA1* (F) or *ZBRK1*. (G) RNAi with or without *GOT* *2* RNAi simultaneously for 48 h. (H) The relative mitochondrial NADH/NAD ^+^ ratio in MCF‐7 cells after transfection with *BRCA1* (right) or *ZBRK1* (left) RNAi with or without *GOT2* RNAi simultaneously for 48 h. Two‐tailed Student's *t*‐test was used to evaluated the difference; error bars represent the mean ± SEM from three independent experiments. ***P < 0.001; **P < 0.01; *P < 0.05; ns, not significant.

Given that GOT2 converts oxaloacetate (OAA) and l‐glutamate (Glu) to generate l‐aspartate (Asp) and 2‐oxoglutarate (also known as α‐ketoglutarate), we next investigated whether BRCA1/ZBRK1 could modulate aspartate metabolism through transcriptionally repressing *GOT2*. It has been reported that GOT2, GPT2 and GDH1 could catalyze glutamate to generate α‐KG (Jin *et al*., 2015); we therefore sought to determine which was predominantly responsible for BRCA1 deficiency. Interestingly, BRCA1 dysregulation did not affect the protein levels of GDH1 and GPT2 in MEF‐BRCA1^▵/▵^ cells compared with the wild‐type counterparts (Fig. S3A). Consistently, knockdown BRCA1 or ZBRK1 in MDA‐MB‐231 cells only increased the protein level of GOT2, not GDH1 or GPT2 (Fig. S3B). We then examined the intracellular and mitochondrial aspartate and α‐KG levels in MEF‐BRCA1^▵/▵^ and MEF‐BRCA1^+/+^ cells. As expected, intracellular and mitochondrial aspartate and α‐KG levels were significantly increased in MEF‐BRCA1^▵/▵^ cells compared with MEF‐BRCA1^+/+^ cells (Fig. 4A,B). Additionally, the mitochondrial NADH/NAD ^+^ redox was significantly elevated in MEF‐BRCA1^▵/▵^ cells compared with MEF‐BRCA1^+/+^ cells (Fig. 4C).To uncover further the underlying mechanisms of BRCA1/ZBRK1 modulation of aspartate biosynthesis, we simultaneously knocked down *BRCA1* with *GOT2* or *ZBRK1* with *GOT2* in MCF‐7 and MDA‐MB‐231 cells, and evaluated the intracellular and mitochondrial Asp and α‐KG levels and NADH/NAD ^+^ redox 48 h after RNAi transfection. As expected, knockdown of *GOT2* in BRCA1 or ZBRK1 downregulated cells reduced the intracellular and mitochondrial aspartate and α‐KG levels compared with BRCA1 or ZBRK1 single knockdown cells, although they did not reach to the level of the control cells (Figs 4D–G and S4A–C). Concordantly, the mitochondrial NADH/NAD ^+^ redox was decreased as well (Fig. 4H,I). This result suggested that the BRCA1/ZBRK1‐GOT2 axis accounted for the Asp and α‐KG production partially and that there another metabolic signaling may be regulated by BRCA1/ZBRK1.

### 3.5 GOT2 mediates metabolic adaptation to promote malignant phenotypes of breast cancer cells

**Figure 5:**
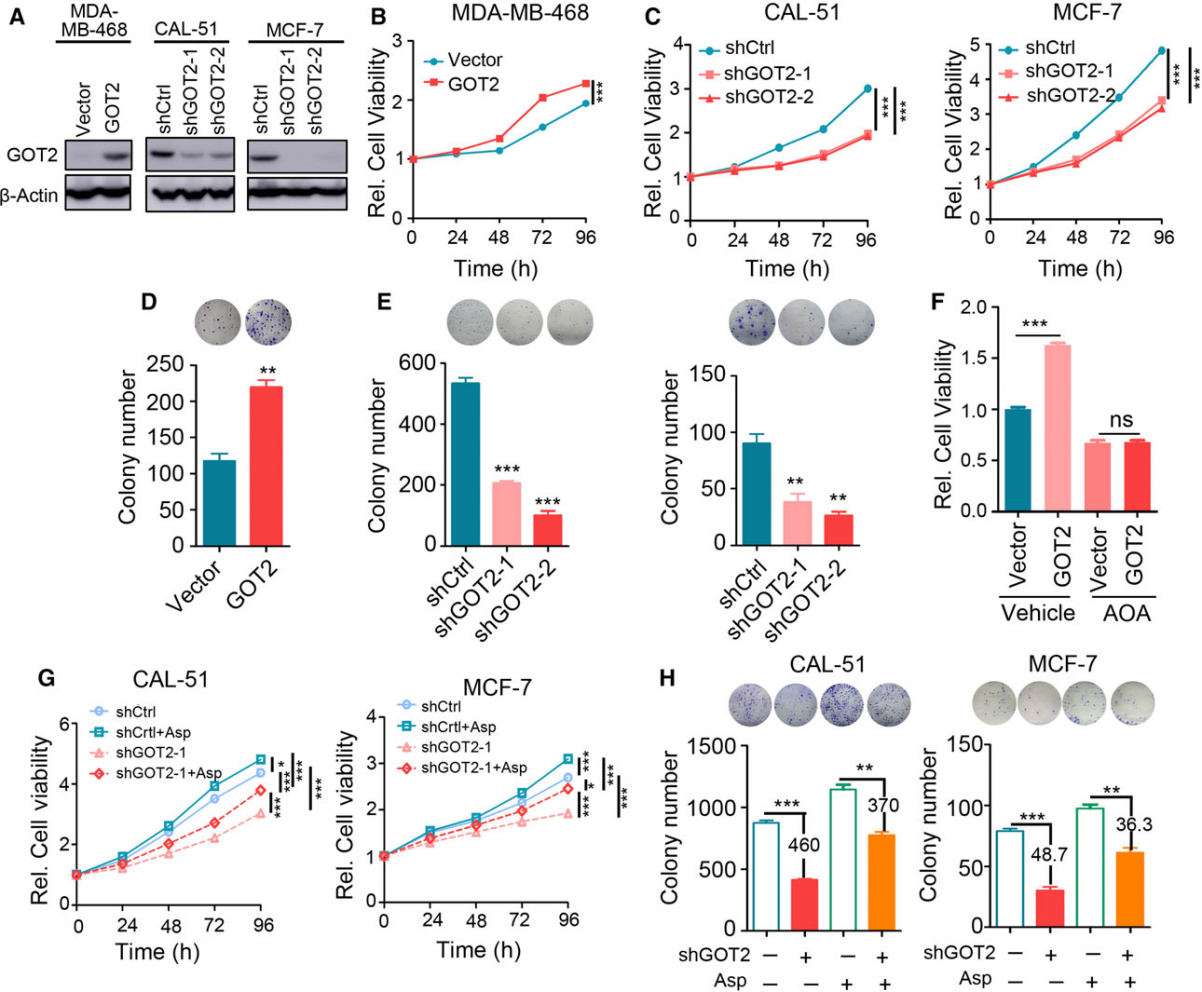
GOT2 promoted breast cancer cells proliferation through generation of aspartate. (A) Western blot analysis of stable ectopic overexpression of GOT2 in MDA‐MB‐468 cells or stable knockdown of GOT2 in CAL‐51 and MCF‐7 cells. (B) Cell proliferation was evaluated in GOT2 overexpressed MDA‐MB‐468 cells and their counterparts. (C) Cell proliferation was evaluated in GOT2 knockdown CAL‐51 or MCF‐7 cells and their counterparts. (D) Colony formation was examined in GOT2 overexpressed MDA‐MB‐468 cells and their counterparts. (E) Colony formation was examined in GOT2 knockdown CAL‐51 or MCF‐7 cells and their counterparts. (F) Cell proliferation was evaluated in GOT2 overexpressed MDA‐MB‐468 cells and their counterparts treated with or without transaminase inhibitor aminooxyacetate (AOA). (G) Cell proliferation was evaluated in GOT2 knockdown CAL‐51 or MCF‐7 cells and their counterparts with or without aspartate. (H) Colony formation was examined in GOT2 knockdown CAL‐51 or MCF‐7 cells and their counterparts with or without aspartate. Two‐tailed Student's *t*‐test was used to evaluated the difference; error bars represent the mean ± SEM from three independent experiments. ***P < 0.001; **P < 0.01; *P < 0.05.

Given that aspartate is a critical metabolite for cell proliferation and biomass production, we further asked whether GOT2 could promote the malignant phenotypes of breast cancer cells through mediating aspartate biosynthesis. Interestingly, analysis using cellminer (http://discover.nci.nih.gov/cellminer) revealed that *GOT2* mRNA expression level was negatively associated with cell doubling time of NCI‐60 cell lines (Pearson *r* = −0.521, *P* < 0.001) or breast cancer cell lines (Pearson *r* = −0.770, *P* = 0.128; Fig. S5A). For further validation, *GOT2* was knocked down or overexpressed via lentiviral infection. We observed that downregulation of GOT2 significantly attenuated, and upregulation of GOT2 promoted, cell proliferation and colony formation of breast cancer cells (Fig. 5A–E). We treated the GOT2 ectopically overexpressed cell line MDA‐MB‐468 and the control cells with aminooxyacetate (AOA), an inhibitor of transaminases, to inhibit the malate‐aspartate shuttle and measured the cell proliferation by MTS assay after 48 h. The results showed that AOA abolished the cell proliferation promotion capacity of GOT2 (Fig. 5F). Given that GOT2 contributes to a portion of aspartate and α‐KG generation, we asked whether adding aspartate could restore the capacities of cell proliferation and colony formation upon GOT2 elimination. Indeed, aspartate addition partially restored the cellular behaviors mentioned above in the context of GOT2 knockdown (Fig. 5G,H). Additionally, we treated GOT2 stable knockdown cell lines CAL‐51 and MCF‐7 and their control cells with α‐KG and found that α‐KG could partially rescue the growth suppression by GOT2 knockdown as well (Fig. 5B,C). Taken together, our results revealed that GOT2 could partially evoke malignant phenotypes of breast cancer cells via its catalytic function.

### 3.6 BRCA1/ZBRK1‐GOT2 axis is associated with clinicopathological characteristics of breast cancer

**Figure 6:**
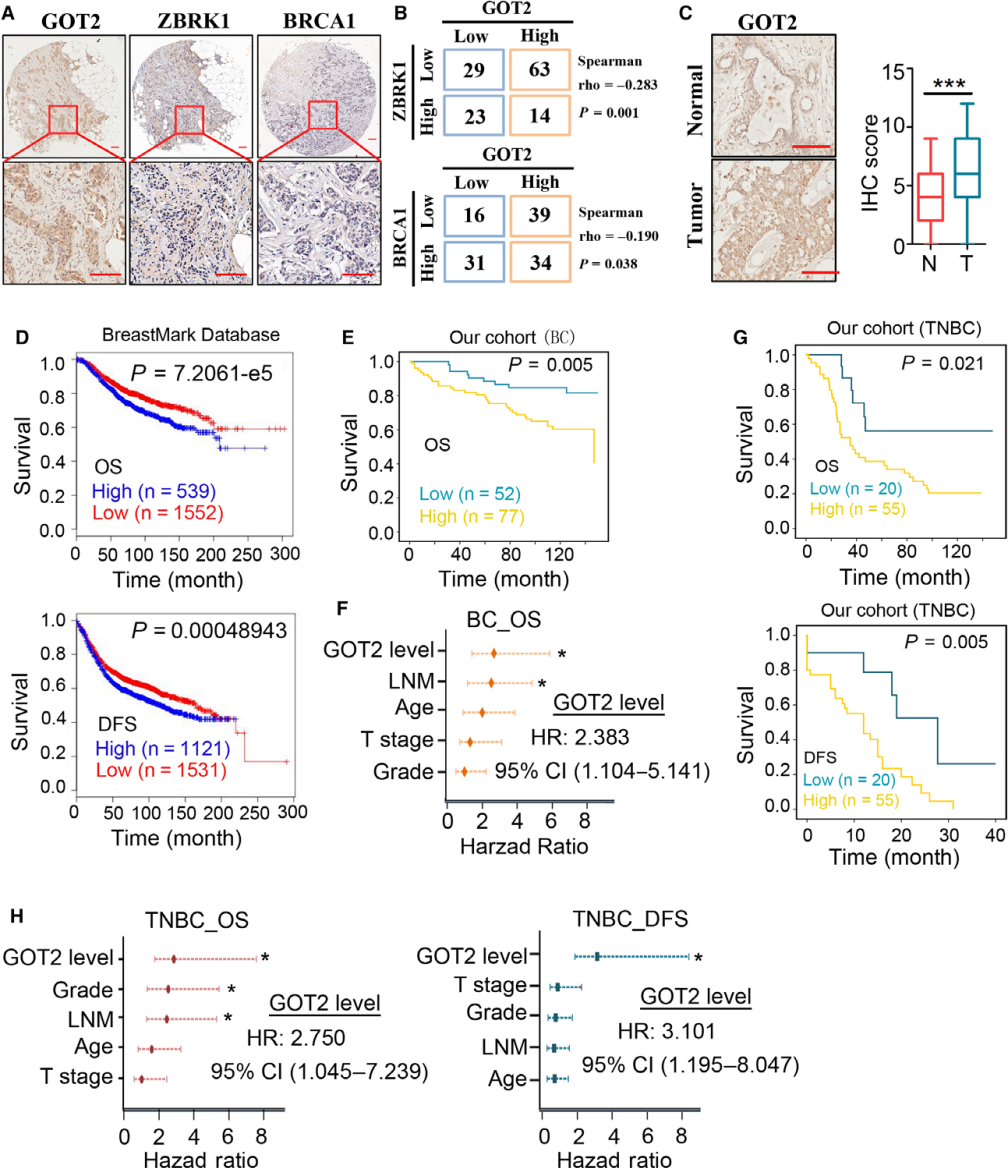
GOT2 overexpression predicted aggressive clinicopathological characteristics and a poor prognosis in breast cancer patients. (A,B) Association between the protein level of GOT2 and BRCA1 or ZBRK1. (A) Representative IHC photos of GOT2, BRCA1 and ZBRK1 in the same breast cancer samples (GOT2 and ZBRK1 were in serial sections, whereas BRCA1 was in the same sample but not the same site, because the serial section TMA from SOBC was sold out). Scale bar: 100 μm. (B) Quantitative data, Pearson chi‐square test and Spearman's rank correlation coefficient analysis were used to determine the correlation between GOT2 and ZBRK1 (Spearman rho = −0.283, *P* = 0.001) or BRCA1 (Spearman rho = −0.190, *P* = 0.038). (C) GOT2 protein level in breast tumor samples (*n* = 130) and adjacent normal tissues (*n* = 90). Left, representative IHC images of GOT2 protein level in breast cancer tissues and adjacent normal tissues. Scale bar: 100 μm. Right, IHC score in breast cancer tissues and adjacent normal tissues. (D) Kaplan–Meier survival analysis (log‐rank test) of BreastMark database cohort stratified by GOT2 mRNA level. Top, overall survival (OS,* n* = 2091, *P* = 7.2061e‐5); bottom, disease‐free survival (DFS,* n* = 2652, *P* = 0.00048943). (E) Kaplan–Meier survival analysis (log‐rank test) of our breast cancer TMA (BC TMA) cohort stratified by GOT2 protein level (*n* = 129, *P* = 0.005). (F) Multivariate Cox analysis of all BC TMA cohorts stratified by different classifiers as indicated. (G) Kaplan–Meier survival analysis (log‐rank test) of triple‐negative breast cancer (TNBC) cohort stratified by GOT2 protein level. Top, overall survival (OS,* n* = 75, *P* = 0.021); bottom, disease‐free survival (DFS,* n* = 75, *P* = 0.005). (H) Multivariate Cox analysis of all TNBC cohorts stratified by different classifiers as indicated. Top, overall survival; bottom, disease‐free survival. ***P < 0.001; *P < 0.05.

We then validated the association between BRCA1/ZBRK1 and GOT2 in BC TMA and another cohort of TNBC tissue samples using immunohistochemistry (IHC) assay. The results revealed that the protein levels of both ZBRK1 and BRCA1 were significantly negative compared with GOT2 in the BC TMA cohort (Spearman's coefficient test, rho = −0.283, *P* = 0.001 and rho = −0.190, *P* = 0.038, respectively) (Fig. 6A,B) and TNBC cohort (Spearman's coefficient test, rho = −0.253, *P* = 0.029 and rho = −0.384, *P* = 0.001, respectively) (Fig. S6A). The protein level of GOT2 was significantly higher in the BC tissue samples than in the adjacent normal tissue samples (Student's test, *P* < 0.001; Fig. 6C). Additionally, the there was a substantially higher frequency of high protein level of GOT2 in the TNBC cohort (73.33%, 55/75) than in the BC cohort (59.69%, 77/129). We next explored the association between GOT2 protein level and different clinicopathological features of patients with BC. In 130 paraffin‐embedded, archived BC TMA cohort, statistical analysis revealed that GOT2 protein level was significantly positively correlated with lymph node metastasis (*P* = 0.039), pathological grade (*P* = 0.019) and American Joint Committee on Cancer (AJCC) stage (*P* = 0.036; Table 1).

**Table 1:** Association of GOT2 protein level with clinicopathological features of 129 breast cancer patients

Clinicopathological features		Total cases (%)	GOT2 protein level		*P*‐value
			High (%)	Low (%)	
Age	≤51	66 (51.2)	39 (59.1)	27 (40.9)	0.887
	> 51	63 (48.8)	38 (60.3)	25 (39.7)	
Lymph node metastasis	YES	41 (32.0)	30 (73.2)	11 (26.8)	**0.039**
	NO	87 (68.0)	47 (54.0)	40 (46.0)	
Grade	I	32 (25.0)	13 (40.6)	19 (59.4)	**0.019**
	II	89 (69.5)	58 (65.2)	31 (34.8)	
	III	7 (5.5)	6 (85.7)	1 (14.3)	
T_Stage	T1	27 (20.9)	17 (63.0)	10 (37.0)	0.697
	T2 + T3	102 (79.1)	60 (58.8)	42 (41.2)	
AJCC_Stage	1 + 2	84 (65.6)	45 (53.6)	39 (46.4)	**0.036**
	3	44 (34.4)	32 (72.7)	12 (27.3)	
Location	Right	72 (55.8)	42 (58.3)	30 (41.7)	0.724
	Left	57 (44.2)	35 (61.4)	22 (38.6)	

The bold *P*‐value indicated the statistical significance.

Notably, the mRNA level of *GOT2* was strongly associated with poor overall survival and disease‐free survival in BC patients in the BreastMark Database cohort (Madden *et al*., 2013) (*P* < 0.001, Kaplan–Meier survival analysis and log‐rank test, Fig. 6D) and KMplot database cohort (Gyorffy *et al*., 2010) (*P* < 0.001 for OS and *P* = 0.034 for DFS, Kaplan–Meier survival analysis and log‐rank test, Fig. S6B), and was also correlated with overall survival of BC patients in the TCGA cohort analyzed by the Human Protein Atlas (https://www.proteinatlas.org/, *P* = 0.015, Fig. S6C). Kaplan–Meier survival analysis of our BC TMA cohort also demonstrated that the protein level of GOT2 was significantly correlated with the poor OS of BC patients (Fig. 6E). Multivariate Cox regression survival analysis adjusting for age, T stage, LNM, pathological grade and GOT protein level consistently reported strong correlation between GOT2 overexpression and shorter survival (*P* = 0.027, Hazard ratio (HR) = 2.383, 95% CI 1.104–5.141) (Fig. 6F, Additional file 1: Table S7), indicating that GOT2 expression was an independent prognostic factor for outcome in BC. In fact, the stratification by GOT2 level displayed even higher prognostic significance than the widely employed LNM (*P* = 0.014, HR = 2.249, 95% CI 1.180–4.285) (Fig. 6F, Table S7). Consistently, the protein level of GOT2 was also strongly associated with poor OS and DFS of TNBC patients (*P* = 0.021 and 0.005, respectively, Kaplan–Meier survival analysis and log‐rank test ) (Fig. 6G). Multivariate Cox regression survival analysis also revealed a strong correlation between GOT2 overexpression and shorter OS and DFS in TNBC cohort (*P* = 0.040, HR = 2.750, 95% CI 1. 045–7.239 for OS and *P* = 0.020, HR = 3.101, 95% CI 1.195–8.047 for DFS) (Fig. 6H, Table S8). Additionally, the mRNA level of GOT2 was significantly positively correlated with poor OS and DFS of Luminal A or HER2‐positive breast cancer and positively correlated with OS in basal‐like subtype BC (Fig. S6D).

Collectively, these findings demonstrated the correlation between BRCA1/ZBRK1 and GOT2 in breast cancer samples and suggested the promising prognostic value of GOT2 in clinical practice.

### 3.7 GOT2 is a potential molecular biomarker indicating the sensitivity of MTX treatment

**Figure 7:**
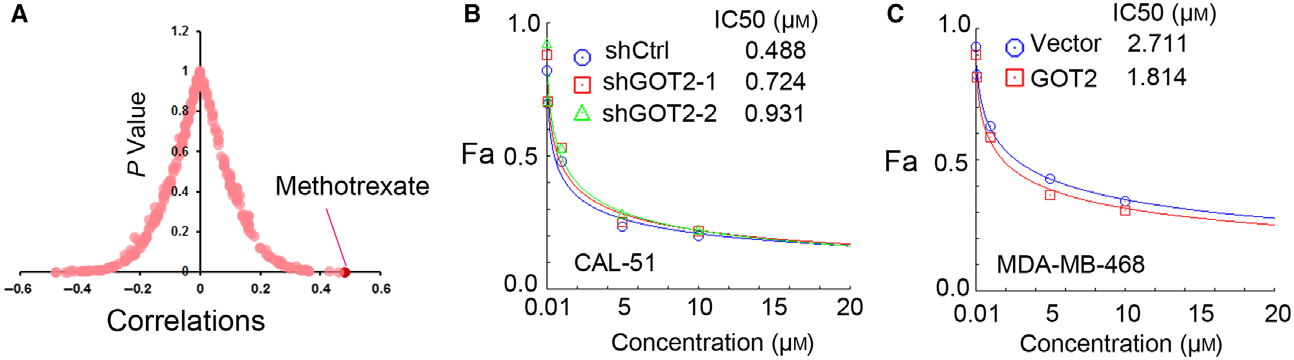
GOT2 expression level is a predictor of methotrexate (MTX) activity. (A) GOT2 mRNA level was significantly positively correlated with MTX activity. (B,C) Dose‐effect curve represent the responses to MTX upon GOT2 knockdown (B) or overexpression (C).

To determine whether GOT2 could serve as a potential molecular biomarker for cancer treatment, we used CellMiner, a web‐based suite of bioinformatics tools designed to explore the drug activity in the NCI‐60 cell lines (Wang *et al*., 2016b) to mine the significantly associated drugs related to the transcription level of *GOT2*. CellMiner tools allow rapid data retrieval of transcripts for 22 379 genes, 92 proteins and 360 microRNA along with activity reports for more than 20 503 chemical compounds, which include 102 drugs approved by the U.S. Food and Drug Administration (FDA). Interestingly, MTX, a classic chemotherapeutic drug for breast cancer treatment, was the top ranked drug positively correlated with GOT2 mRNA level among FDA approved drugs or those under clinical trials (Fig. 7A, Table S9). However, the expression level of ZBRK1 was weakly negatively correlated with the drug activity of MTX (*r* = −0.226, *P* = 0.0832; Table S10), and BRCA1 showed no significant correlation with the drug activity of MTX (*r* = 0.214, *P* = 0.100) (Table S11). For further validation, we treated breast cancer cells that were stable ectopic overexpression or knockdown of GOT2 with different concentrations of MTX. The results showed that knockdown of GOT2 substantially increased the IC_50_ of MTX, whereas overexpression of GOT2 reduced the IC_50_ in breast cancer cells (Fig. 7B,C). These findings suggested that GOT2 expression might act as a predictor of the MTX activity.

## 4 Discussion

**Figure 8:**
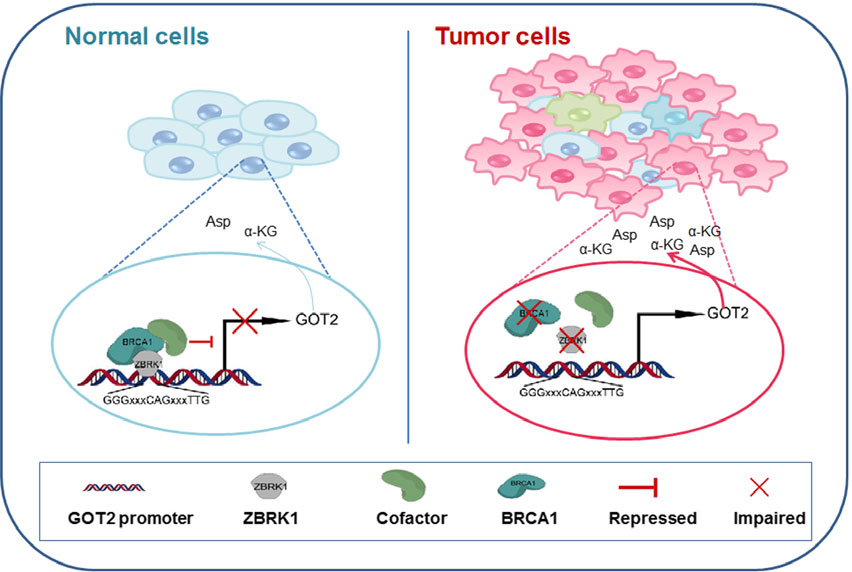
Schematic representation of the BRCA1/ZBRK1‐GOT2 proposed mode of action in modulating breast tumor responses. In normal mammary cells, BRCA1 and ZBRK1 forms a co‐repressor complex on the promoter of *GOT2* and suppresses its transcription, whereas in BRCA1‐ or ZBRK1‐deficient breast tumor cells, impairment of BRCA1/ZBRK1 co‐repressor complex fails to inhibit the transcription of *GOT2* and thus leads to GOT2 upregulation and subsequently accelerated aspartate and α‐KG production.

BRCA1 has been implicated with cell metabolism in previous studies (Privat *et al*., 2014); however, the underlying mechanisms are still unclear. As a transcriptional co‐repressor, there is still no direct evidence to support the notion that BRCA1 mediates tumor metabolic adaptation through its transcriptional function. In the present study, we demonstrated for the first time that BRCA1 formed a co‐repressor complex with ZBRK1 on the promoter of GOT2 via the ZBRK1 recognition element, and that BRCA1 deficiency promoted aspartate and α‐KG production and accelerated tumor cell growth along with GOT2 upregulation (Fig. 8). Importantly, we found that GOT2 was frequently overexpressed in BC, especially in TNBC, which was significantly associated with poor survival of patients with these diseases and could serve as an independent prognostic biomarker. These findings indicated that GOT2 was a malignant metabolic driver in the absence of BRCA1 or ZBRK1 in BC. Interestingly, our preliminary results revealed that GOT2‐overexpressed BC cells were more sensitive to MTX treatment, suggesting a promising therapeutic strategy for precision treatment of BC patients.

Depletion of BRCA1 in mouse embryonic fibroblast induced differentiated expression of a dozen metabolic genes, and a majority of these genes contained the consensus ZBRK1 DNA‐binding element, indicating that BRCA1 and ZBRK1 share common regulation pathways in metabolism modulation. BRCA1 has been reported to form a co‐repressor complex with ZBRK1 to suppress the expression of many genes, including *GADD45A* (Yun and Lee, 2003), *HMGA2* (Ahmed *et al*., 2010), *MMP9* (Lin *et al*., 2010) and *ANG1* (Furuta *et al*., 2006). However, whether this co‐repressor complex participates in metabolism regulation is still unclear. Strikingly, our results provided the first evidence that aspartate transaminase GOT2 was a direct target of BRCA1/ZBRK1. A series of observations supported this conclusion. First, GOT2 was upregulated both in BRCA1 depletion or knockdown cells and in ZBRK1 knockdown cells. Secondly, ChIP assay using antibodies against BRCA1 or ZBRK1 revealed that BRCA1 and ZBRK1 co‐occupied the *GOT2* promoter. Moreover, by ChIP/re‐ChIP assay analysis, BRCA1 and ZRBK1 were further confirmed to form a complex on the *GOT2* promoter. Thirdly, luciferase reporter assay verified that BRCA1 and ZBRK1 exerted transcriptional repression activity on *GOT2* expression. Deletion of the ZBRK1 recognizing motif GGGxxxCAGxxxTTG of *GOT2* abolished the transcriptional repression effect of BRCA1 ectopic expression, further suggesting that BRCA1 regulated the expression of *GOT2* through ZBRK1. A previous study has demonstrated that CtIP formed a complex with BRCA1 and ZBRK1 to repress the expression of ANG1 (Furuta *et al*., 2006). It seems that BRCA1 and CtIP facilitate ZBRK1 binding to the recognition site *in vivo* (Tan *et al*., 2004); however, whether CtIP is essential for the regulation of *GOT2* by BRCA1/ZBRK1 remains to be elucidated.

Interestingly, the expression of BRCA1 was previously reported to be controlled by the NAD^+^/NADH ratio (Di *et al*., 2010). An increased NAD^+^/NADH ratio led to disassociation of HDAC1 from BRCA1 promoter, thus promoting BRCA1 expression. Here, we showed that BRCA1 deficiency resulted in transcriptional activation of *GOT2*, concomitantly leading to increased NADH/NAD ^+^ redox. Accordingly, it may be speculated that GOT2 overexpression can cause BRCA1 downregulation and subsequently influence the canonical functions of BRCA1. The potential feed‐forward loop of BRCA1/ZBRK1/GOT2 may be a crucial metabolic driver signaling BC progression. Recently, GOT2 was shown to activate transcriptionally STAT3/NF‐κB in IL‐10 responsiveness (Feist *et al*., 2018). One may propose that GOT2 is fine‐tuned with different cell metabolic status and different stimuli in the tumor microenvironment. The specific context and the mechanisms of how the repressor complex is removed from the ZBRK1 site, and how these two pathways are coordinated in regulating *GOT2* expression, warrant further investigation.

Aspartate is required for rapid cell growth, which is the most important intermediate for protein, purine nucleotide and pyrimidine nucleotide synthesis (Chen *et al*., 1996; Sullivan *et al*., 2015). Given that aspartate has one of the lowest levels of all circulating amino acids (Mayers and Vander Heiden, 2015), obtaining aspartate from the blood for tumor cells is a great challenge. Thus aspartate synthesis is essential for cancer progression. Actually, aspartate metabolism reprogramming exists in many cancers, including hepatocellular carcinoma (Darpolor *et al*., 2014), pancreatic cancer (Yang *et al*., 2015, 2018), leukemia (Sullivan *et al*., 2015), lung cancer, cervical cancer, osteosarcoma and glioblastoma (Sullivan *et al*., 2015). GOT2 converts glutamate and oxaloacetate to generate aspartate and α‐KG, which is the critical enzyme for maintaining the aspartate pool (Feist *et al*., 2018). Previous studies have revealed that a crucial role of cellular respiration is to maintain redox homeostasis to support aspartate and therefore biomass production and rapid proliferation (Birsoy *et al*., 2015; Sullivan *et al*., 2015). Coloff *et al*. (2016) demonstrated that mammary epithelial cells can switch from oxidative deamination of Glu to α‐KG catalyzed by GDH1 to the conversion of OAA into aspartate by transamination of Gln catalyzed by GOT. Interestingly, Hui Yang *et al*. elucidated a novel mechanism whereby SIRT3‐dependent GOT2 acetylation affects the malate–aspartate NADH shuttle activity and promotes pancreatic tumor growth (Yang *et al*., 2015). Additionally, GOT2 overexpression could inhibit senescence of pancreatic tumor cells and therefore contribute to tumor progression (Yang *et al*., 2018). Thus, it is reasonable to propose that GOT2 is a metabolic hub, providing cells with aspartate and/or α‐KG for energy demands and biosynthetic processes. In our present study, we showed that BRCA1 or ZBRK1 deficiency resulted in GOT2 overexpression, and enhanced the aspartate and α‐KG production, subsequently promoting cell proliferation. These observations provided a novel perspective for understanding how BRCA1 and ZBRK1 contribute to BC progression. However, simultaneous knockdown of GOT2 and BRCA1 or ZBRK1 did not completely restore aspartate and α‐KG to basal level. This discrepancy indicates that BRCA1/ZBRK1 might also coordinate aspartate and α‐KG metabolism by other metabolic pathways, and is worthy of further investigation.

GOT2 has not been associated with the sensitivity of MTX in previous studies. Here, we found that GOT2 overexpression sensitized breast cancer cells to MTX. GOT2 is a key enzyme providing aspartate for nucleotide synthesis, which sustains rapid cell proliferation (Feist *et al*., 2018). It is considered that MTX treatment causes cancer cells death mainly by reducing the cellular pool of tetrahydrofolate (THF), which inhibits nucleotide synthesis (Hitchings and Burchall, 1965; Kanarek *et al*., 2018). It is probable that cancer cells with high GOT2 expression are addictive in nucleotide synthesis, supporting rapid cell proliferation and thus leading to THF decline. Recently, Naama Kanarek and colleagues reported that histidine ammonia lyase (HAL), the rate‐limiting enzyme of the histidine degradation pathway, contributed to the sensitivity of cancer cells to MTX by competitive consumption of THF (Kanarek *et al*., 2018). Interestingly, data mined by CellMiner revealed that histidine degradation‐related gene *HAL* and nucleotide synthesis‐related genes *IMPDH2*,* MTHFD1*,* PFAS*,* PPAT* and *ATIC* were significantly coexpressed with *GOT2* (data not show). Therefore, we proposed that GOT2 overexpression might be coupled with activation of histidine degradation pathway and nucleotide synthesis pathways to decrease the cellular level of THF, thus sensitized breast cancer cells to MTX.

## 5 Conclusions

Our study provides evidence that BRCA1 and ZBRK1 possess an additional role in tumor suppression, regulating the aspartate metabolism through transcriptional repression of GOT2. Importantly, our data reveal that GOT2 can serve as an independent prognostic factor for survival outcome of patients with BC and a potential predictor for sensitivity of MTX treatment. These findings extend our knowledge of tumor suppressive functions of BRCA1, providing a new perspective on metabolism regulation and raising the possibility that synthetically targeting aspartate metabolism may be a promising strategy for treatment of BRCA1‐deficient breast tumors.

## Conflict of interest

The authors declare no conflict of interest.

## Author contributions

QZ, SZ, RH and WZ designed the study. RH, WZ, XX, KZ, YW, MW, JF, JL, WX performed experiments. WZ provided computational analysis. FX and JC provided expert consultation. RH and WZ wrote the manuscript. QZ and SZ supervised the study.

## Supporting information


**Fig. S1.** ASS1 was regulated by BRCA1 but not ZBRK1.
**Fig. S2.** Chromatin immunoprecipitation (ChIP) of RNA polymeraseII in MCF‐7 cells.
**Fig. S3.** GDH1 and GPT2 were not regulated by BRCA1 or ZBRK1.
**Fig. S4.** BRCA1 and ZBRK1 affected the protein level of GOT2 and the mitochondrial α‐KG level.
**Fig. S5.** GOT2 promoted cell proliferation.
**Fig. S6.** GOT2 associated with clinicopathological characteristics.

**Table S1.** Significant differentially expression of metabolic genes in MEF BRCA1^▵/▵^ and MEF BRCA1^+/+^ cell lines.
**Table S2.** BRCA1 repressed metabolic pathways.
**Table S3.** BRCA1 upregulated metabolic pathways.
**Table S4.** Survival analysis of the potential ZBRK1 transcriptional repressed metabolic genes by BreastMark database.
**Table S5.** The clinicopathological characteristics of 130 breast cancer samples.
**Table S6.** The clinicopathological characteristics of 75 triple‐negative breast cancer samples.
**Table S7.** Multivariate COX regression analysis for the identification of prognostic factors for overall survival in the GOT2 IHC BC TMA cohort.
**Table S8‐1.** Multivariate COX regression analysis for the identification of prognostic factors for overall survival in the GOT2 IHC TNBC cohort.
**Table S8‐2.** Multivariate COX regression analysis for the identification of prognostic factors for disease‐free survival in the GOT2 IHC TNBC cohort.
**Table S9.** Correlations between the GOT2 mRNA level and drug activities.
**Table S10.** Correlations between the ZBRK1 mRNA level and drug activities.
**Table S11.** Correlations between the BRCA1 mRNA level and drug activities.
