# Corrigendum to: Preventing BRCA1/ZBRK1 repressor complex binding to the *GOT2* promoter results in accelerated aspartate biosynthesis and promotion of cell proliferation

**DOI:** 10.1002/1878-0261.12784

**Published:** 2020-09-10

The authors of Hong *et al*. [1] omitted to state the species each primer set was intended for, which could mislead efforts to reproduce the results. To clarify the species in which each primer set was used, authors have provided the following amendment to the Methods section below. This change does not affect any conclusions resulting from this study.

## 2.3 RNA isolation and quantitative real‐time PCR

Total RNAs of breast cancer cells and mouse embryonic fibroblasts MEF BRCA1^+/+^ and MEF BRCA1^Δ/Δ^ were extracted with TRIzol reagent (Invitrogen, Carlsbad, CA, USA). The cDNA was synthesized with the PrimeScript RT reagent Kit (Promega, Madison, WI, USA). Real‐time PCR was carried out using an ABI 7500 real‐time PCR system (Applied Biosystems, Foster City, CA, USA). The mRNA expression level of GOT2 and ASS1 was normalized to the endogenous expression of GAPDH. Primers were provided by Invitrogen as described below:

**Table:** 

Mouse
GAPDH Forward, 5′‐ GTTGTCTCCTGCGACTTCA ‐3′
GAPDH Reverse, 5′‐ TGGTCCAGGGTTTCTTACTC ‐3′
GOT2 Forward, 5′‐ GAGCAGGGCATCAATGTCTG ‐3′
GOT2 Reverse, 5′‐ GTTGGAATACAGGGGACGGA ‐3′
ASS1 Forward, 5′‐ ACCATCCTTTACCACGCTCA ‐3′
ASS1 Reverse, 5′‐ CGCTCCTGGGACTTCTGGATA ‐3′
Human
GAPDH Forward, 5′‐ TCTCTGCTCCTCCTGTTC ‐3′
GAPDH Reverse, 5′‐ GTTGACTCCGACCTTCAC ‐3′
GOT2 Forward, 5′‐ TTACGTTCTGCCTAGCGTCC ‐3′
GOT2 Reverse, 5′‐ ACTTCGCTGTTCTCACCCAG ‐3′
ASS1 forward, 5′‐ TGTGCTTATAACCTGGGATGGG ‐3′
ASS1 reverse, 5′‐ GACATAGCGTCTGGGATTGGA ‐3′
