# Supplementary material for: Preventing BRCA1/ZBRK1 repressor complex binding to the GOT2 promoter results in accelerated aspartate biosynthesis and promotion of cell proliferation
Source: Mol Oncol. 2019 Mar 1;13(4):959–77. doi: 10.1002/1878-0261.12466 (PMC6441895; doi:10.1002/1878-0261.12466)
Supplement: Supplementary file 1 — Fig. S1. ASS1 was regulated by BRCA1 but not ZBRK1. Fig. S2. Chromatin immunoprecipitation (ChIP) of RNA polymeraseII in MCF‐7 cells. Fig. S3. GDH1 and GPT2 were not regulated by BRCA1 or ZBRK1. Fig. S4. BRCA1 and ZBRK1 affected the protein level of GOT2 and the mitochondrial α‐KG level. Fig. S5. GOT2 promoted cell proliferation. Fig. S6. GOT2 associated with clinicopathological characteristics. [file MOL2-13-959-s001.docx]

**Supplementary figures and figure legends**

**
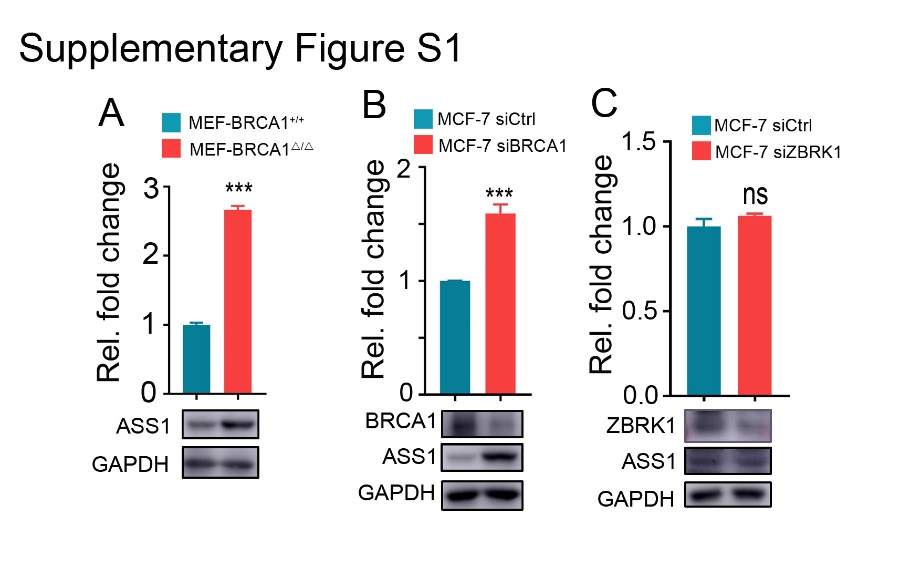
**

**Supplementary Figure S1 ASS1 was regulated by BRCA1 but not ZBRK1. (A)**, The mRNA (top) and protein level (bottom) of ASS1 in MEF-BRCA1^△/△^ cells and MEF-BRCA1^+/+^ cells. **(B, C)**, The mRNA (top) and protein level (bottom) of ASS1 in MCF-7 cells after knockdown of BRCA1 (B) or ZBRK1 (C) for 48h. Two tailed Student’s t-test was used to evaluated the difference, error bars represent the mean ± S.E.M from three independent experiments, ***p < 0.001. ns, not significant.


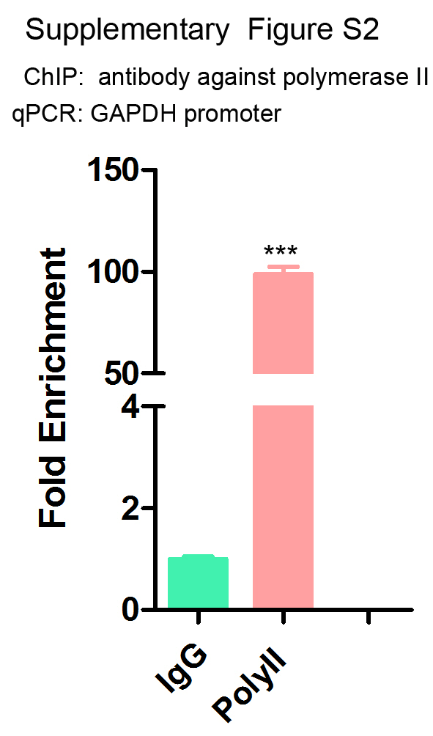


**Supplementary Figure S2 Chromatin immunoprecipitation (ChIP) of RNA polymeraseⅡ in MCF-7 cells.** Displayed are average qPCR results and technical errors (S.E.M.) of the GAPDH promoter. Fold enrichment was calculated relative to IgG. Two tailed Student’s t-test was used to evaluated the difference, error bars represent the mean ± S.E.M from three independent experiments, ***p < 0.001.


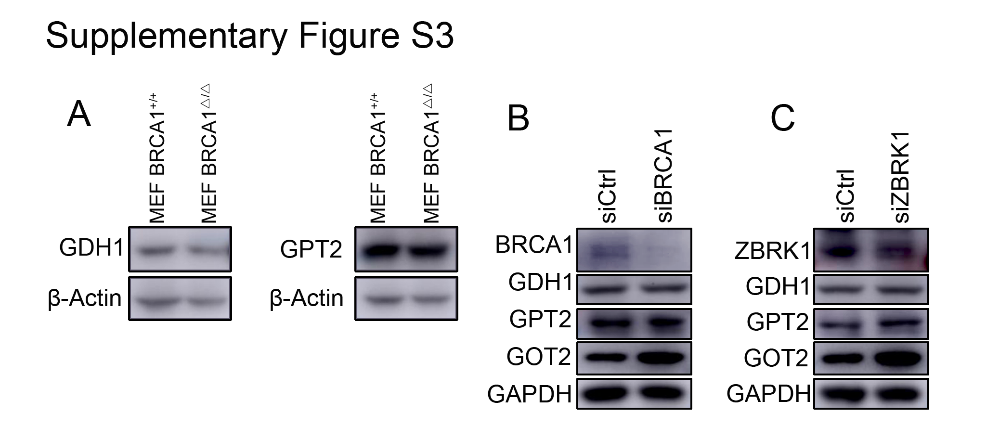


**Supplementary Figure S3 GDH1 and GPT2 were not regulated by BRCA1 or ZBRK1** (A), The protein level of GDH1 and GPT2 in MEF-BRCA1^△/△^ cells and MEF-BRCA1^+/+^ cells. (B, C), The protein levels of GDH1, GPT2 and GOT2 in MDA-MB-231 cells after knockdown of BRCA1 (B) or ZBRK1 (C) for 48h.

**
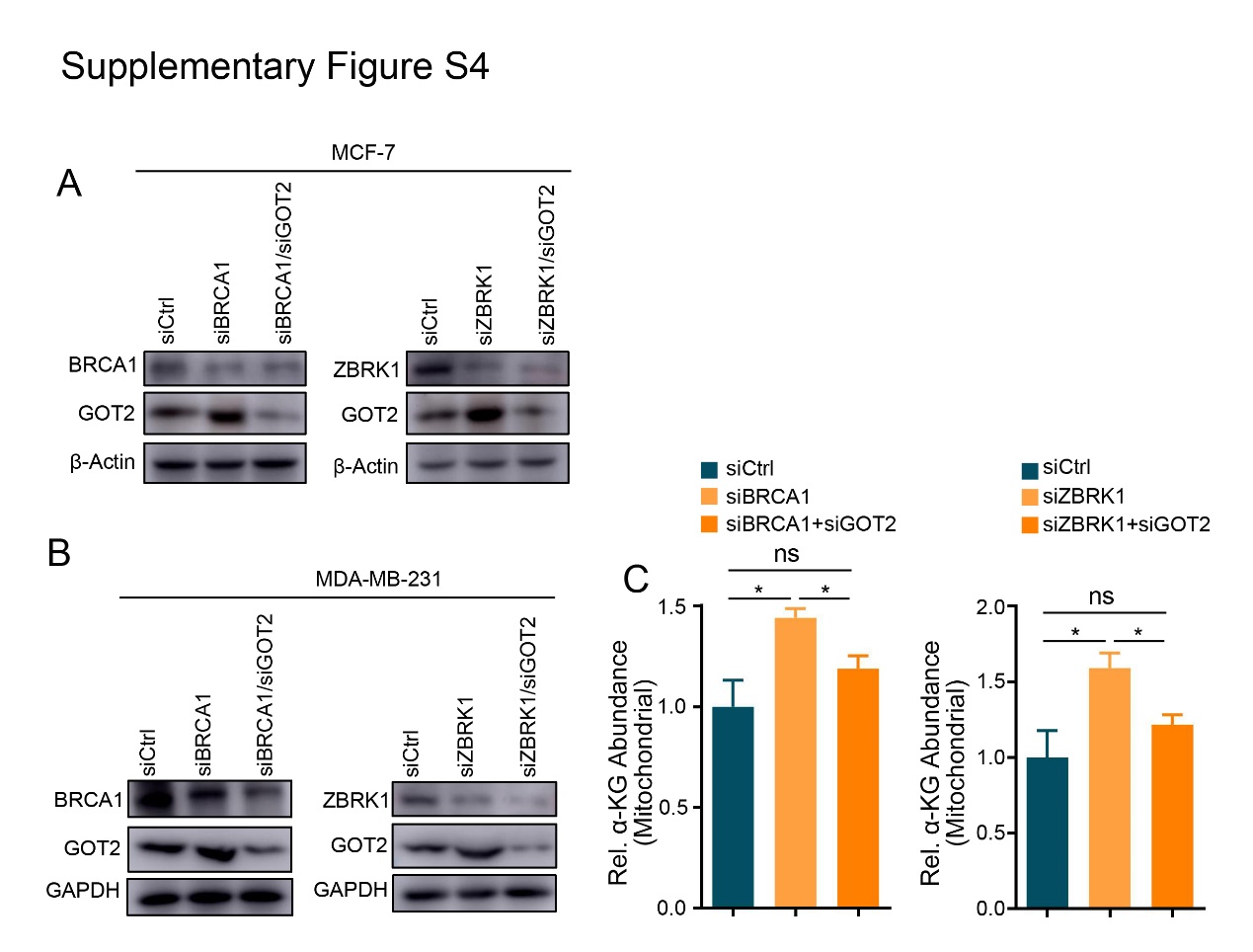
**

**Supplementary Figure S4 BRCA1 and ZBRK1 affected the protein level of GOT2 and the mitochondrial α-KG level.** (A), Western blot examined BRCA1, ZBRK1 and GOT2 protein levels in MCF-7 (A) and MDA-MB-231 (B) after transfected with BRCA1 and ZBRK1 RNAi along or with GOT2 RNAi respectively for 48h. (C), The relative abundance of mitochondrial α-KG in MDA-MB-231 cells after transfected with *BRCA1* (Right) or *ZBRK1* (Left) RNAi without or with *GOT2* RNAi simultaneously for 48h. Two tailed Student’s t-test was used to evaluated the difference, error bars represent the mean ± S.E.M from three independent experiments, *p < 0.05. ns, not significant.

**
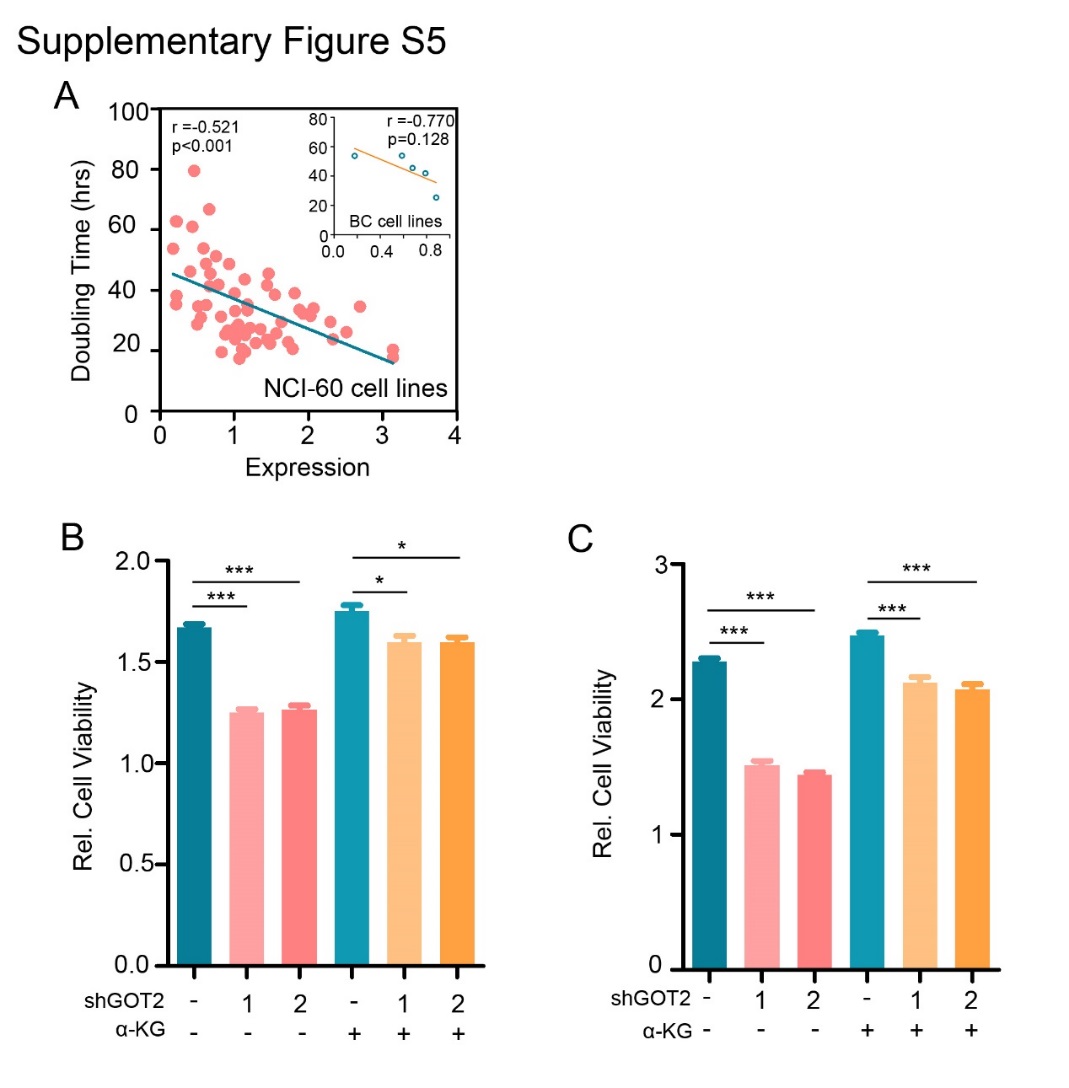
**

**Supplementary Figure S5 GOT2 promoted cell proliferation.** (A) Correlations between GOT2 mRNA level and cell doubling time among NCI-60 cells and five breast cancer cells. (B, C), Cell proliferation was evaluated in GOT2 knockdown CAL-51 (B) or MCF-7 (C) cells and their counterparts with or without 2 mM α-KG for 48h. 1, means shGOT2-1, 2, means shGOT2-2. Two tailed Student’s t-test was used to evaluated the difference, error bars represent the mean ± S.E.M from three independent experiments, ***p < 0.001. *p < 0.05.

**
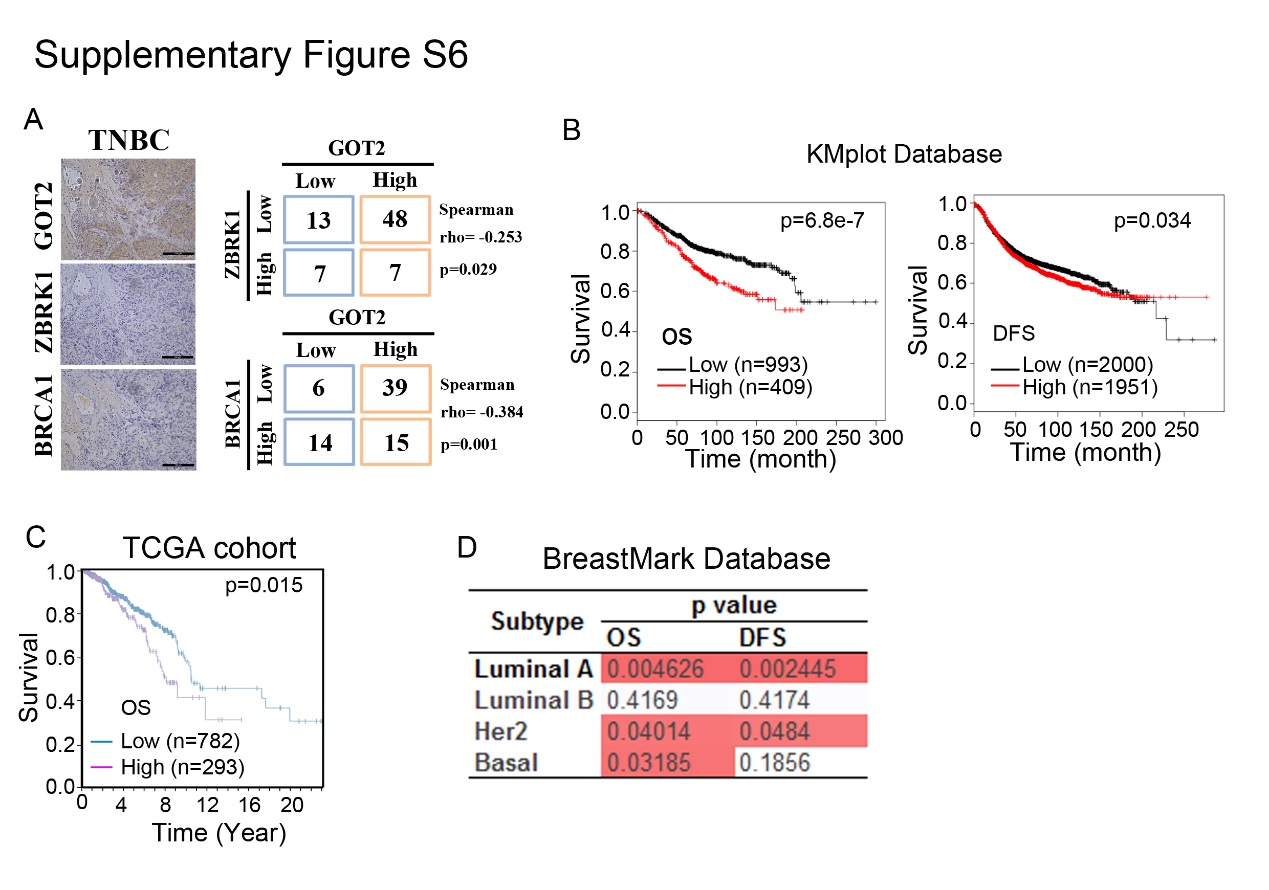
**

**Supplementary Figure S6 GOT2 associated with clinicopathological characteristics. (A)**, Association between the protein level of GOT2 and BRCA1 or ZBRK1 in TNBC samples. Left, Representative IHC photos of GOT2, BRCA1 and ZBRK1 in the same TNBC case. Scale bar: 100 μm. Right, quantitative data, Pearson χ2 test and Spearman's rank correlation coefficient analysis were used to determine the correlation between GOT2 and ZBRK1 (Spearman rho=-0.253, p=0.029) or BRCA1 (Spearman rho=-0.384, p=0.001). **(B)**, Kaplan-Meier survival analysis (log-rank test) of KMplot database cohort stratified by GOT2 mRNA level. Left, Overall survival (OS, n=1402, p=6.8e-6); Right, Disease free survival (DFS, n=2951, p=0.034). **(C)**, Kaplan-Meier survival analysis (log-rank test) of TCGA cohort stratified by GOT2 mRNA level (OS, n=1075, p=0.015). **(D)**, Kaplan-Meier survival analysis (log-rank test) of different subtypes of breast cancer by BreastMark Web tool stratified by GOT2 mRNA level.
